# On the Downlink Capacity of Cell-Free Massive MIMO with Constrained Fronthaul Capacity

**DOI:** 10.3390/e22040418

**Published:** 2020-04-07

**Authors:** Peng Zhang, Frans M. J. Willems

**Affiliations:** 1 IMEC the Netherlands, 5656AE Eindhoven, The Netherlands; 2 Department of Electrical Engineering, Technical University of Eindhoven, 5612AZ Eindhoven, The Netherlands; f.m.j.willems@tue.nl

**Keywords:** channel capacity, distributed beamforming, cell-free MIMO, constrained fronthaul

## Abstract

We investigate the downlink of a cell-free massive multiple-in multiple-out system in which all access points (APs) are connected in a linear-topolpgy fronthaul with constrained capacity and send a common message to a single receiver. By modeling the system as an extension of the multiple-access channel with partially cooperating encoders, we derive the channel capacity of the two-AP setting and then extend the results to arbitrary *N*-AP scenarios. By developing a cooperating mode concept, we investigate the optimal cooperation among the encoders (APs) when we limit the total fronthaul capacity, and the total transmit power is constrained as well. It is demonstrated that achieving capacity requires a water-pouring distribution of the total available fronthaul capacity over the fronthaul links. Our study reveals that a linear growth of total fronthaul capacity results in a logarithmic growth of the beamforming capacity. Moreover, even if the number of APs would be unlimited, only a finite number of them need to be activated. We found an expression for this number.

## 1. Introduction

Recently, cell-free massive multiple-input multiple-output (mMIMO) has been considered as a key technology for beyond-5G networks. In such user-centric transmission systems, a large number of distributed access points (APs) are connected to one central processing unit (CPU) via fronthaul links and phase coherently cooperate to cover a wide area for a small number of users in the same time-frequency resource using time-division operation. Compared to cell-based collocated mMIMO solutions, such technology improves energy-spectral efficiency and enhances immunity to shadow fading without extra signal processing burdens. We refer to [1,2,3] and the references therein for a general overview of current developments of cell-free mMIMO.

Effectively utilizing fronthaul resources is of critical importance for deploying a scalable cell-free mMIMO system. Considering the downlink for instance, simple distributed conjugate beamforming is optimal, as shown in [1]. However, it can already be seen that a large amount of information exchange over fronthaul links is required since all the APs need to know the message that is to be transmitted. A star-topology fronthaul where each APs are individually connected to a CPU was originally modeled and has been widely studied, see, e.g., [4,5,6] and the references therein. Currently, a serial fronthaul connecting APs in a linear topology is considered for achieving a cost-efficient architecture, both in deployment and maintenance [3]. A novel and promising technique relying on a linear topology is the radio stripe system, where multiple APs are embedded in a cable/strip, see [3,7] in detail. Such radio stripes can be easily and invisibly deployed indoor or outdoor in existing constructions to enable numerous new applications [8].

**Figure 1:**
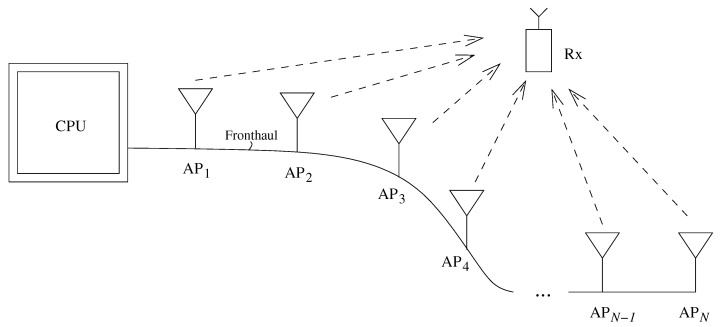
Cell-free transmit beamforming where access points (APs) are connected via serial fronthaul.

The focus of prior work in cell-free mMIMO study was on developing wireless signaling techniques. In this paper, we study from an information-theoretic perspective the downlink of a cell-free mMIMO system shown by Figure 1, where single-antenna APs are connected in a linear topology with constrained fronthaul capacities to communicate to one single-antenna terminal receiver (Rx).

The considered multiple-in single-out (MISO) setup forms a distributed massive beamforming system and can be formulated as a multiple-access-channel (MAC) with limited fronthaul capacity, which is defined as the maximal amount of information that can be reliably sent per MAC channel use [9]. By investigating the channel capacity of such a MAC, we reveal essential relations between the three the most fundamental resources of the system, i.e., the total available number of APs (*N*), the total transmit power (*P*), and total available fronthaul capacity ($C_{B}$). Specifically, in the current cell-free mMIMO literature, the only configuration of APs that is considered is where full cooperation (full beamforming) is realized and where the same information is shared at all involved APs. Therefore, for a real-valued Gaussian MISO channel with *N* APs and unity channel gains, the maximum downlink rate is given by the channel capacity

(1) $$\begin{array}{c} C_{\mathrm{full}}:=\frac{1}{2}\log_{2}\left(1+N\cdot \mathrm{SNR}\right)\;\;\;\;\;\mathrm{bits}/\mathrm{channel}\;\mathrm{use} \end{array}$$

where SNR is the received signal-to-noise ratio (SNR) if only one AP is active with all available transmit power assigned to it. It requires $C_{B}^{\mathrm{full}}:=\left(N-1\right)C_{\mathrm{full}}$ fronthaul capacity among *N* APs.

In this work, we focus on the case where the available fronthaul capacity is not large enough to support full cooperation of the APs. We were motivated to investigate the achievable downlink rates given that fronthaul resources for communication between the APs is constrained. We call this setting partial beamforming, since $C_{B}<C_{B}^{\mathrm{full}}$. We could derive the channel capacity and the optimal cooperation strategies among APs for given total available *P* and *N*.

### 1.1. Related Work

We can model the studied system as a special extension of the multiple-access channel (MAC) with partially cooperating encoders studied by Willems [9]. In particular, we can generalize the system setup in [9] to a network of encoders by considering only one source but employing an arbitrary number of encoders, namely APs, via unidirectional conferences. Since fronthaul links can be treated as separate channels that are orthogonal to the beamforming MAC, our setup might also be viewed as an extension of a special case of the orthogonal-component relay channel due to El Gamal and Zahedi [10], which is generalized to relay networks by Ghabeli and Aref in [11]. In addition, if only two APs are considered, our study is also strongly related to the multiple access diamond channel as studied in [12,13]. Moreover, the two APs setup looks very similar to the semi-deterministic relay channels [14]. Furthermore, it is also worth to note that in our system, all APs cooperatively send one message to a receiver at a same time. In this sense, our channel setting is “noncausal”, which is related to the relay-with-delay channel studied in [15] in general.

### 1.2. Contributions and Organization

By investigating the MAC with limited fronthaul capacity in the discrete channel case and in the Gaussian channel cases, the main findings of our research work include

- The channel capacity is found for an arbitrary number of APs for both discrete channel and the Gaussian channel with constrained transit power, where the total fronthaul capacity and the total number of APs are limited.
- When numerous APs are engaged, a linear growth of total fronthaul capacity results in a logarithmic growing of the channel (beamforming) capacity.
- A concept of cooperating modes is developed to demonstrate the optimal cooperation among APs to achieve capacity based on superposition coding.
- When the channel capacity is only limited by the fronthaul capacity, the number of required APs is quasi-linear to the available fronthaul capacity even if the number of APs would be unlimited.
- A new and sharp lower bound of the Lambert-W function is derived for computing the number of required APs given by the total fronthaul constraint.

In the rest of this paper, the system model is first presented in Section 2. In Section 3, we start with investigating a two-APs setting consisting of one fronthaul link. This setting serves as a baseline system where the cooperating mode concept is developed. In Section 4, the study is extended to the case where an arbitrary number of APs is engaged and the behavior and exact solution of the channel capacity is derived. In Section 5, the number of required APs is derived to leverage limited fronthaul resources if the number of available APs is unlimited. Finally, the conclusion and final remarks can be found in Section 6. Detailed proofs and derivations of the presented results are collected in the Appendix A. Partial material in this paper was presented in [16].

## 2. Problem Setup

### 2.1. Notation

Although all the paper, capital letters, e.g., *X*, denote random variables, and their realizations are denoted by small letters, e.g., *x*. The probability mass or density function according to *X* is denoted by $p_{X}\left(x\right)$ or simply p(x). The expectation of *X* is denoted by E[X]. The entropy of *X* is denoted by H(X) and the differential entropy is denoted by h(X). The mutual information between *X* and *Y* is denoted by I(X;Y). The consecutive integer range from *i* to *j* with i≤j is denoted by [i:j]. In addition, a set of elements $x_{m}$ with index *m* in range of *i* to *j* is denoted as ${\left\{x_{m}\right\}}_{m=i}^{j}$.

### 2.2. System Model

**Figure 2:**
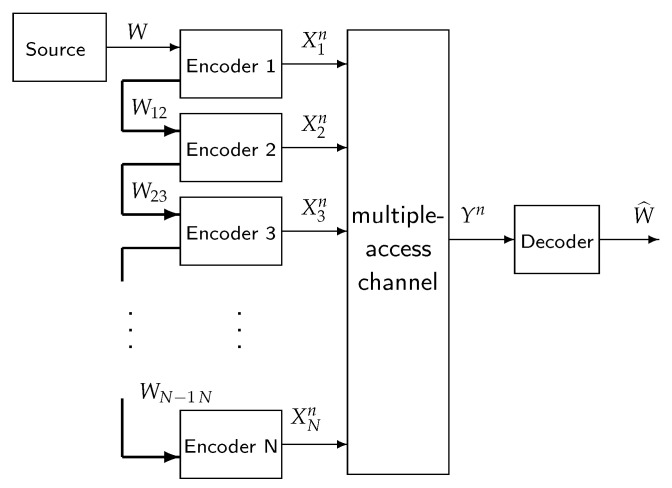
*N* encoders that cooperate in sending a message *W* to a decoder with limited fronthaul communication.

The investigated system is modeled as Figure 2, where we denote the CPU as the source, the APs as encoders, while for the destination, the receiver is denoted as the decoder. As plotted, one-directional fronthaul links connect *N* adjacent encoders that simultaneously send a uniformly distributed message W∈[1:M] to a decoder (receiver). We focus on the study of the fronthaul resource usage among all encoders. The discrete memoryless MAC denoted by $\left(X_{1}\times X_{2}\times \ldots \times X_{N},p\left(y|x_{1},x_{2},\ldots ,x_{N}\right),Y,{\left\{C_{m,m+1}\right\}}_{m=1}^{N-1}\right)$ consists of input alphabets ${\left\{X_{m}\right\}}_{m=1}^{N}$, output alphabet Y, a transition probability distribution $p(y|x_{1},x_{2},\ldots ,x_{N})$, and a set of fronthaul capacity constraints ${\left\{C_{m,m+1}\right\}}_{m=1}^{N-1}$ between *N* encoders.

Before the beginning of each *n* channel uses, (partial) information about the generated message *W* is first shared among *N* encoders. Let $W_{m,m+1}\in \left[1:M_{m,m+1}\right]$ for m∈[1:N−1] be the message sent over the fronthaul link between encoder *m* to encoder m+1. Then, the encoders map the messages *W* and ${\left\{W_{m,m+1}\right\}}_{m=1}^{N-1}$ into codewords ${\left\{x_{m}^{n}\right\}}_{m=1}^{N}$ as follows

$$\begin{array}{ccc} e_{1}\left(W\right) & \to & (X_{1}^{n},W_{12}),\\ e_{m}\left(W_{m-1,m}\right) & \to & (X_{m}^{n},W_{m,m+1}),\\ e_{N}\left(W_{N-1,N}\right) & \to & X_{N}^{n}, \end{array}$$

where ${\left\{e_{m}\left(\cdot \right)\right\}}_{m=1}^{N}$ are the corresponding encoding functions. Meanwhile, the generated fronthaul messages should satisfy

(2) $$\frac{1}{n}\log_{2}M_{m,m+1}\leq C_{m,m+1}.$$

As presented, the corresponding fronthaul link capacity $C_{m,m+1}\geq 0$ is defined as the maximal amount of information that can be reliably sent per channel use of the MAC channel over the link from encoder *m* to encoder m+1.

At the decoder, a deterministic decoding function $d:Y^{n}\to \left[1:M\right]$ is applied to obtain the message-estimate $\hat{W}$ based on the channel output $y^{n}$. We define the average probability of error at the decoder as

(3) $$P_{e}^{\left(n\right)}\overset{\Delta }{=}\mathrm{Pr}\left(\hat{W}\neq W\right).$$

Now we say that a rate *R* is achievable with given fronthaul capacities ${\left\{C_{m\;m+1}\right\}}_{m=1}^{N-1}$ if there exists *N* encoders and a corresponding decoder, such that

(4) $$\begin{array}{ccc} \log_{2}M & \geq & n(R-\delta ),\\ \log_{2}M_{m,m+1} & \leq & nC_{m,m+1},\\ P_{e}^{\left(n\right)} & \leq & \delta , \end{array}$$

for all δ>0 and large enough *n*. The channel capacity *C* (of MAC) as a function of the fronthaul capacities is defined as the supremum of all achievable rates given by all the fronthaul constraints. Eventually, we will be interested only in a constraint on the sum of the fronthaul capacities $C_{B}$ that is defined as

(5) $$C_{B}\overset{\Delta }{=}\sum_{m=1}^{N-1}C_{m,m+1}.$$

To interpret the capacity results for the partial beamforming, we focus on MACs with additive white Gaussian noise. At the output of the Gaussian MAC, the decoder receives

(6) $$Y_{i}=\sum_{m=1}^{N}X_{mi}+Z_{i},$$

at time *i*, where $X_{mi}$ is the transmitted symbol by encoder *m* and $Z_{i}$ is modeled as independent and identically distributed (i.i.d.) Gaussian noise at the decoder for all i∈[1:n]. For individual encoder *m*, m∈[1:N], the transmit power constraint is

(7) $$\frac{1}{n}\sum_{i=1}^{n}E\left[X_{mi}^{2}\right]\leq P_{m}$$

for $P_{m}\geq 0$. Then, the total transmit power is limited as

(8) $$\sum_{m=1}^{N}P_{m}\leq P.$$

Without loss of generality, we assume that $Z_{i}∼N\left(0,1\right)$. Therefore, the transmit SNR can be directly represented by the total constrained transmit power *P*.

## 3. Two-Encoder Result

We first investigate the simplest system setting where only two encoders are involved. The MAC is now denoted by $\left(X_{1}\times X_{2},p\left(y|x_{1},x_{2}\right),Y,C_{12}\right)$. The fronthaul message $W_{12}\in \left[1:M_{12}\right]$ must satisfy the constraint

(9) $$\frac{1}{n}\log_{2}M_{12}\leq C_{12},$$

which is same to the total fronthaul capacity $C_{B}$ in this case. The underlying Gaussian MAC is given by

(10) $$Y_{i}=X_{1i}+X_{2i}+Z_{i}.$$

Although this two-encoder setting can be considered as a special case of related work, see discussion later, we provide here the capacity proofs for both discrete and Gaussian MACs. The applied approach carries over to the *N*-encoder setting that is investigated in Section 2.

In the following, the channel capacity as a function of the fronthaul capacity is first obtained for the discrete memoryless MAC. Then, we derive capacity results for the Gaussian case with total transmit power constraint. Within this study, a so-called *cooperating mode* concept is developed that will be very useful to provide cooperation insights among encoders when more of them are engaged.

### 3.1. Discrete Channel

First, consider the discrete channel setup.

**Theorem** **1.** *For the discrete memoryless channel $p(y|x_{1},x_{2})$, the channel capacity C as a function of the fronthaul capacity $C_{12}$ is given by*

(11) $$C\left(C_{12}\right)=\max_{p(x_{1},x_{2})}\min\left\{I\left(X_{1},X_{2};Y\right),I\left(X_{1};Y|X_{2}\right)+C_{12}\right\},$$

*where distribution $p\left(x_{1},x_{2},y\right)=p\left(x_{1},x_{2}\right)p\left(y|x_{1},x_{2}\right)$ is determined by the input distribution $p(x_{1},x_{2})$.*

The detailed proof is provided in Section Appendix A.1, where the converse is based on the Markovities of $W\to \left(X_{1}^{n},X_{2}^{n}\right)\to Y^{n}$ and $\left(W,W_{12}\right)\to \left(X_{1}^{n},X_{2}^{n}\right)\to Y^{n}$, and the achievability is based on applying superposition coding. For the achievability, the source splits the message *W* into two parts $\left(W_{1},W_{12}\right)$ and delivers the index of $W_{12}$ over the fronthaul link to encoder 2 that maps $W_{12}$ into the inner code while encoder 1 of the source encodes $W_{1}$ into an outer code-word which is super-imposed on the inner code-word. Although this coding scheme is simple, the *cooperating mode* concept that is important for studying the multi-encoder setup will be developed based on the superposition scheme as discussed later.

**Remark** **1.** *By viewing the two-encoder setting as a special setup of ([9], Figure 1), where only one source and one conference link are deployed, we can have Theorem 1 by letting the common message $U=X_{2}$ and the conference capacity $C_{21}=0$ in ([9], Thm.). Note that the achievability in [9] which is based on binning becomes superposition coding.*

**Remark** **2.** *By viewing the two-encoder setting as a special setup of the multiple access diamond channel where one source connects to two encoders (relays) by using two separate noiseless links, see [12,13], Theorem 1 can also be obtained if letting $C_{1}=\infty$, the common message $V=X_{2}$, and the common message rate $R_{0}=C_{2}$ (or $C_{2}=\infty$, $V=X_{1}$, $R_{0}=C_{1}$) in ([12], Thm. 2). Note that the achievability based on superposition and Marton-coding in [12,13] becomes superposition coding only.*

### 3.2. Gaussian Channel

Now we consider the Gaussian MAC of the two-encoder channel setting given by (10) with total power constraint *P*, i.e., $P_{1}+P_{2}\leq P$, where

(12) $$\frac{1}{n}\sum_{i=1}^{n}E\left[x_{1i}^{2}\right]\leq P_{1}\;\;\;\mathrm{and}\;\;\;\frac{1}{n}\sum_{i=2}^{n}E\left[x_{2i}^{2}\right]\leq P_{2}.$$

This first leads to the following result.

**Theorem** **2.** *The channel capacity $C(C_{12},P)$ of the two-encoder Gaussian MAC is*

(13) $$\begin{array}{ccc} C(C_{12},P) & = & \max_{0\leq \beta \leq 1}\min\left\{\frac{1}{2}\log_{2}\left(1+\left(1+\beta \right)P\right),\frac{1}{2}\log_{2}\left(1+\left(1-\beta \right)P\right)+C_{12}\right\}. \end{array}$$

The proof is the adaptation of the discrete channel version given in Section Appendix A.1 by considering the transmit power constraints and Gaussian channel noise.

**Proof.**  (i) *Converse*. First note that without loss of generality (and without violating the power constraints) we may assume that all $E\left[X_{1i}\right]=E\left[X_{2i}\right]=0$ for all i∈[1,n]. If we define $\left(X_{1},X_{2},Y\right)$ being the random triple with density $p_{X_{1},X_{2},Y}\left(x_{1},x_{2},y\right)=\frac{1}{N}\sum_{i=1}^{N}p_{X_{1i},X_{2i},Y_{i}}\left(x_{1},x_{2},y\right)$ then converse in Section Appendix A.1 shows that

(14) $$\begin{array}{ccc} I(X_{1}^{n},X_{2}^{n};Y^{n}) & \leq & nI(X_{1},X_{2};Y),\\ I(X_{1}^{n};Y^{n}|X_{2}^{n}) & \leq & nI(X_{1};Y|X_{2}), \end{array}$$

where the random variables $X_{1}$, $X_{2}$ satisfy $E\left[X_{1}\right]=E\left[X_{2}\right]=0$ and

(15) $$\begin{array}{ccc} E[X_{1}^{2}] & \leq & \frac{1}{n}\sum_{i=1}^{n}E\left[X_{1i}^{2}\right]\leq P_{1}, \end{array}$$

(16) $$\begin{array}{ccc} E[X_{2}^{2}] & \leq & \frac{1}{n}\sum_{i=1}^{n}E\left[X_{2i}^{2}\right]\leq P_{2}. \end{array}$$

First consider the random pair $\left(X_{1},X_{2}\right)$. By applying the Cholesky factorization ([17], Thm. 4.2.7) to the covariance matrix of ${\left[X_{2},X_{1}\right]}^{T}$, the assignment of

(17) $$\left\{\begin{array}{lll} X_{1} & = & \alpha_{21}S_{2}+\alpha_{1}S_{1},\\ X_{2} & = & \alpha_{22}S_{2}, \end{array}\right.$$

can be obtained, where $S_{1}$ and $S_{2}$ are uncorrelated with zero means and unit variances.

Next, observe that if we take $\alpha_{21}^{\prime }=\alpha_{22}^{\prime }=\left(\alpha_{21}+\alpha_{22}\right)/2=\alpha_{2}$ this choice does not affect $I\left(X_{2};Y\right)=I\left(S_{2};Y\right)$ and $I\left(X_{1};Y|X_{2}\right)=I\left(S_{1};Y|S_{2}\right)$, but minimizes the total transmit power for fixed $\alpha_{21}+\alpha_{22}$, since

(18) $$2\alpha_{2}^{2}=2{\left(\frac{\alpha_{21}+\alpha_{22}}{2}\right)}^{2}\leq \alpha_{21}^{2}+\alpha_{22}^{2}.$$

Therefore we only need to consider assignment

(19) $$\left\{\begin{array}{lll} X_{1} & = & \alpha_{2}S_{2}+\alpha_{1}S_{1},\\ X_{2} & = & \alpha_{2}S_{2}. \end{array}\right.$$

Now we take $\alpha_{2}^{2}=P_{2}$, $\alpha_{2}^{2}+\alpha_{1}^{2}=P_{1}$, and $2\alpha_{2}^{2}+\alpha_{1}^{2}=P$. By denoting

(20) $$\beta \overset{\Delta }{=}\frac{2\alpha_{2}^{2}}{P}$$

in [0,1], we further have

(21) $$\alpha_{1}^{2}=\left(1-\beta P\right)\;\;\;\mathrm{and}\;\;\;\alpha_{2}^{2}=\beta P/2.$$

Taking the signal assignment (19) and the power assignment (21) gives that

(22) $$\begin{array}{ccc} I(X_{1},X_{2};Y) & = & I\left(S_{1},S_{2};Y\right)=h\left(\alpha_{1}S_{1}+2\alpha_{2}S_{2}+Z\right)-h\left(Z\right)\\ & \overset{\left(a\right)}{\leq } & \frac{1}{2}\log_{2}\left(1+\left(1+\beta \right)P\right), \end{array}$$

(23) $$\begin{array}{ccc} I(X_{1};Y|X_{2}) & = & I\left(S_{1};Y|S_{2}\right)=h\left(\alpha_{1}S+Z\right)-h\left(Z\right)\\ & \overset{\left(b\right)}{\leq } & \frac{1}{2}\log_{2}\left(1+\left(1-\beta \right)P\right), \end{array}$$

where (a) and (b) follow by the maximum differential entropy theorem, see ([18], Thm. 8.6.5).

(ii) *Achievability*. Taking the assignment (19) by letting $S_{1}∼N\left(0,1\right)$ and $S_{2}∼N\left(0,1\right)$. Using the power assignment (21) directly gives

(24) $$\begin{array}{ccc} I(X_{1},X_{2};Y) & = & I\left(S_{1},S_{2},Y\right)=\frac{1}{2}\log_{2}\left(1+\left(1+\beta \right)P\right), \end{array}$$

(25) $$\begin{array}{ccc} I(X_{1};Y|X_{2}) & = & I\left(S_{1};Y|S_{2}\right)=\frac{1}{2}\log_{2}\left(1+\left(1-\beta \right)P\right). \end{array}$$

The rest of the proof follows by first establishing a coding theorem for the discrete memoryless channel with input cost (power constraint). The step from discrete to Gaussian channels is justified by the relation between differential entropy and discrete entropy, see, e.g., ([18], Thm. 9.3.1). □

Now, by optimizing over β in (13), we can further express *C* as a function only in total transmit power *P* and total fronthaul capacity $C_{B}$, which is $C_{12}$ for this two-encoder setup.

**Corollary** **1.** *The channel capacity $C(C_{B},P)$ of the total transmit power constrained two-encoder Gaussian MAC can be expressed as*

(26) $$\begin{array}{c} C(C_{B},P)=\left\{\begin{array}{c} \frac{1}{2}\log_{2}\left(1+2P\right),\;\;\;\mathrm{if}\;\;\;C_{B}\geq \frac{1}{2}\log_{2}\left(1+2P\right)\;\\ \frac{1}{2}\log_{2}\left(1+P\right)+\frac{1}{2}\log_{2}\left(2-\frac{2}{2^{2C_{B}}+1}\right),\;\;\;\;\mathrm{otherwise}. \end{array}\right. \end{array}$$

**Proof.**  The two logarithms on the RHS of  (13) are monotonically increasing and decreasing in β respectively and equal to each other at β=0. Hence, we can set

(27) $$\frac{1}{2}\log_{2}\left(1+\left(1+\beta \right)P\right)=\frac{1}{2}\log_{2}\left(1+\left(1-\beta \right)P\right)+C_{B}$$

to obtain the β that maximizes *C* for $\forall C_{B}\in \left[0,\frac{1}{2}\log_{2}\left(1+2P\right)\right]$ as

(28) $$\beta^{⋆}=\frac{\left(1+P\right)\left(2^{2C_{B}}-1\right)}{P(2^{2C_{B}}+1)}.$$

This results in the second capacity expression in (26). Then, if $C_{B}>\frac{1}{2}\log_{2}\left(1+2P\right)$, the second term is always larger than the first term for any β in (13). This corresponds to the situation where $C_{B}$ is large enough and the transmission over the MAC is the bottleneck of the network. In this case, *C* remains at its global maximum. □

Note that, for $C_{B}<\frac{1}{2}\log_{2}\left(1+2P\right)$, the first term of the capacity result (26) is the channel capacity with no beamforming and the second term directly represents the partial beamforming gain that is independent of transmit power *P* and only grows as the fronthaul capacity increases. As revealed, the partial beamforming gain increases with a same rate regardless of the transmit power *P*.

### 3.3. Cooperating Modes

Based on assignment (19) that possesses a superposition structure, we can naturally denote two *cooperating modes* as what follows to describe the optimal cooperation between the encoders for the capacity achieving.

- *mode 1*: Sending a private message given by $\alpha_{1}S_{1}$ from encoder 1;
- *mode 2*: Coherently sending a common message given by $\alpha_{2}S_{2}$ from encoder 2 and encoder 1.

According to (20), the parameter β represents the fraction of the total transmit power assigned to *mode* 2 while 1−β represents the remaining fraction assigned to *mode* 1. Note that $\beta^{⋆}$ given by (28) should be taken for achieving the capacity.

**Figure 3:**
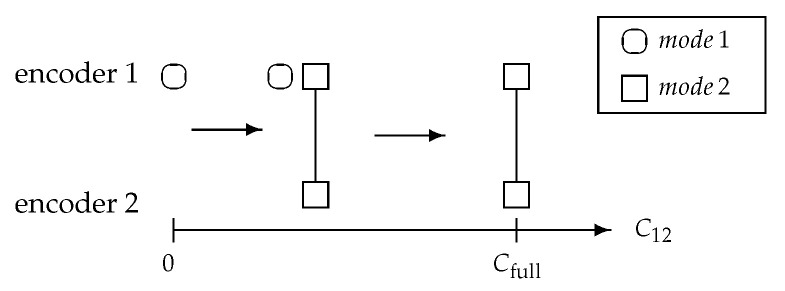
Modes activation for the two-encoder Gaussian setting.

Now consider the cooperation scenarios of the two encoders based on the availability of $C_{B}$. If $C_{B}=0$, the transmission reduces to the point-to-point communication case. This is represented by having only mode 1 active and encoder 2 is inactive. If $C_{B}\geq C_{\mathrm{full}}$, full cooperation can be achieved by activating mode 2 only. For $C_{12}\in \left(0,C_{\mathrm{full}}\right)$, two encoders cooperate to achieve partial beamforming capacity by activating both *cooperating modes*. Figure 3 illustrates the *cooperating modes* activating and deactivating at encoders depending on $C_{B}$ increasing from 0 to $C_{\mathrm{full}}$.

For the two-encoder setting, the modes evolution due to available amount of $C_{B}$ looks straightforward. Nevertheless, it will be shown that this *cooperating modes* interpretation provides a clear insight of leveraging available encoders for given certain total fronthaul and transmit power constraints, where the optimal cooperation is not trivial as the number of encoders goes largely.

## 4. *N*-Encoder Result

Based on the investigations of the two-encoder setting, we extend the study to the system model with arbitrarily *N* encoders, where N≥2. The parameter *N* in principle can be any large integer so that a distributed massive beamforming is obtained. The investigation is focused on the Gaussian MAC under the constraints of the total fronthaul capacity $C_{B}$ and the total transmit power *P*, which are defined by (5) and (8), respectively. Before addressing the exact capacity solution for arbitrary *N* encoders, we first derive capacity bounds of $C(C_{B},P)$ to provide a general behavior of channel capacity *C* in total fronthaul capacity $C_{B}$. The obtained result indicates that the growth of *C* requires an exponential growth of $C_{B}$. By using the *compound mode*, the exact capacity solution with the optimal cooperation among encoders are derived. The results show that the distributed beamforming system works most efficiently when it is working in its fronthaul-capacity-limited regime. As a result, we consider the case where encoders are always available to be activated as needed to leverage the entire fronthaul resource.

### 4.1. Discrete Channel

For simplicity, let the tuple ${\underline{X}}_{l}^{m}≜\left(X_{l},X_{l+1},\ldots ,X_{m}\right)$ be the collection of ordered transmitted random variables that are generated at encoder *l* to encoder *m* with l≤m for one channel use. In addition, let ${\underline{C}}_{b}≜{\left\{C_{j,j+1}\right\}}_{j=1}^{N-1}$ be the collection of the corresponding fronthaul capacities.

**Theorem** **3.** *For the discrete memoryless N-encoder setting, channel capacity C of the channel $P(y|x_{1},x_{2},\ldots ,x_{N})$ as a function of fronthaul capacities ${\underline{C}}_{b}$ is*

(29) $$C\left({\underline{C}}_{b}\right)=\max_{p(x_{1},x_{2},\ldots ,x_{N})}\min\left\{I\left({\underline{X}}_{1}^{N};Y\right),I\left({\underline{X}}_{1}^{m};Y|{\underline{X}}_{m+1}^{N}\right)+C_{m,m+1}{\}}_{m=1}^{N-1}\right\},$$

*with N≥2.*

A sketch of the proof is given in Section Appendix A.2. As shown in the achievability, the capacity is achieved by applying an *N*-layer superposition coding among the encoders, which naturally agrees with the studied linear topology.

### 4.2. Gaussian Channel under Total fronthaul Constraint

By considering on the total power and separate fronthaul constraints, we first have the following result.

**Theorem** **4.** *The the N-encoder Gaussian setting with the total transmit power constraint of P, the channel capacity C as a function of the fronthaul capacities ${\underline{C}}_{b}$ is*

(30) $$\begin{array}{c} C\left({\underline{C}}_{b},P\right)=\max_{\underline{\beta }}\min\left\{\frac{1}{2}\log_{2}\left(1+\sum_{l=1}^{N}l\beta_{l}P\right),{\left\{\frac{1}{2}\log_{2}\left(1+\sum_{l=1}^{m}l\beta_{l}P\right)+C_{m,m+1}\right\}}_{m=1}^{N-1}\right\}, \end{array}$$

*where $\underline{\beta }={\left(\beta_{1},\beta_{2},\ldots ,\beta_{N}\right)}^{T}$ is a probability vector.*

**Proof.**  Similar to proof of the two-encoder setting, the generic signal assignment

(31) $$X_{m}=\sum_{l=m}^{N}\alpha_{lm}S_{l}$$

can be used at each encoder for m∈[1,N], where ${\left\{S_{l}\right\}}_{l=1}^{N}$ are uncorrelated and have zero mean and unit variance. Again, we can further apply the special signal assignment

(32) $$X_{m}=\sum_{l=m}^{N}\alpha_{l}S_{l}$$

to minimize the total transmit power without affecting dependency of the different signals ${\left\{S_{l}\right\}}_{l=1}^{N}$ at the decoder that determines the beamforming capacity. In this way, the transmit power allocated for signal $S_{l}$ can be expressed by

(33) $$\beta_{l}=\frac{l\alpha_{l}^{2}}{P}$$

such that $\sum_{l=1}^{N}\beta_{l}=1$.

Thus, for the converse, we can use the assignment  (32) to evaluate (29) and the mutual informations on the RHS are bounded as given by (30). Then, for the achievability, by letting $S_{l}∼N\left(0,1\right)$, the result follows. □

The proof shows that all the transmitted signals at encoders should form a Markov chain $X_{N}\to X_{N-1}\to \cdots \to X_{1}$. Again, since signal $\alpha_{l}S_{l}$ represents the common messages used at first *l* encoders, we say that *cooperating mode* *l* is active if the signal $S_{l}$ is generated and sent and there can be *N* *cooperating modes* in total for this *N*-encoder setting.

Now, we can solve the optimization problem

(34) $$\begin{array}{cccc} \; & \underset{{\underline{C}}_{b}}{\mathrm{maximize}} & \; & C({\underline{C}}_{b},P) \end{array}$$

(35) $$\begin{array}{cccc} \; & subject to\; & \; & \sum_{m=1}^{N-1}C_{m,m+1}=C_{B}, \end{array}$$

where $C({\underline{C}}_{b},P)$ is given by (30), to investigate the total power limited capacity *C* under the constraint of total fronthaul capacity $C_{B}$ for a given *P*. To do so, we first prove the following lemma. Note that the full-cooperation capacity is now $C_{\mathrm{full}}\left(N\right)=\frac{1}{2}\log_{2}\left(1+NP\right)$ when *N* encoders are used. For simplicity, we denote the mutual informations as

(36) $$I_{m}≜\frac{1}{2}\log_{2}\left(1+\sum_{l=1}^{m}l\beta_{l}P\right)$$

for any m∈[1:N].

**Lemma** **1.** *For the N-encoder setting with any given $C_{B}\leq \left(N-1\right)C_{\mathrm{full}}\left(N\right)$, power distribution $\underline{\beta }$ can only be optimal if equality of all the terms on the RHS of (30) is achieved.*

The proof is given in Appendix A.3.

**Remark** **3.** *Lemma 1 indicates that asymmetric distribution of $C_{B}$ over fronthaul link is optimal. This result will be further demonstrated after the capacity result is derived.*

Based on the reduced $\underline{\beta }$ set given by Lemma 1, we make the terms on the RHS of (30) equal and have

(37) $$C_{m,m+1}=I_{N}-I_{m}\;\;\;\;\mathrm{with}\;\;\;m\in \left[1:N-1\right].$$

Thus, the channel capacity and the required total fronthaul capacity in the power allocation vector $\underline{\beta }$ can now be represented as

(38) $$\begin{array}{c} C\left(\underline{\beta },P\right)=I_{N}=\frac{1}{2}\log_{2}\left(1+\sum_{l=1}^{N}l\beta_{l}P\right), \end{array}$$

and

(39) $$\begin{array}{c} C_{B}\left(\underline{\beta },P\right)=\left(N-1\right)I_{N}-\sum_{m=1}^{N-1}I_{m}=\frac{N-1}{2}\log_{2}\left(1+\sum_{l=1}^{N}l\beta_{l}P\right)-\sum_{m=1}^{N-1}\frac{1}{2}\log_{2}\left(1+\sum_{l=1}^{m}l\beta_{l}P\right) \end{array}$$

respectively for a fixed *P* if $C_{B}\leq \left(N-1\right)C_{\mathrm{full}}\left(N\right)$. Based on (38) and (39), we can have the following theorem.

**Theorem** **5.** *For the N-encoder Gaussian channel under the total power constraint P and total fronthaul constraint $C_{B}$, the channel capacity is given by*

$$\begin{array}{cccc} \; & \underset{\underline{\beta }}{\mathrm{maximize}} & \; & C\left(\underline{\beta },P\right)=I_{N} \end{array}$$

(40) $$\begin{array}{cccc} \; & subject to\; & \; & \left(N-1\right)I_{N}-\sum_{m=1}^{N-1}I_{m}=C_{B} \end{array}$$

(41) $$\begin{array}{ccc} \; & \; & \sum_{l=1}^{N}\beta_{l}=1\;\;\mathrm{and}\;\;0\leq \beta_{l}\leq 1. \end{array}$$

To evaluate the channel capacity, we only need to maximize the function by introducing a Lagrange multiplier λ as

(42) $$\begin{array}{ccc} g(\underline{\beta },P,\lambda ) & = & I_{N}-\lambda C_{B}\\ \; & = & \left(1-\left(N-1\right)\lambda \right)I_{N}+\lambda \sum_{m=1}^{N-1}I_{m}\\ \; & = & \frac{1-(N-1)\lambda }{2}\log_{2}\left(1+\sum_{l=1}^{N}l\beta_{l}P\right)+\frac{\lambda }{2}\sum_{m=1}^{N-1}\log_{2}\left(1+\sum_{l=1}^{m}l\beta_{l}P\right) \end{array}$$

under the constraint that $\underline{\beta }$ is a probability vector to derive the solution of $C(C_{B})$ for the general *N*-encoder case. Note that the parameter λ is the slope of $C(C_{B})$. However, before working out the exact solution of this optimization problem, we first derive general bounds of $C(C_{B},P)$ to reveal the capacity behavior of the studied distributed beamforming.

### 4.3. Capacity Behavior Bounds

To obtain a simple but meaningful insight of the relation between *C* and the constrained $C_{B}$ and *P* for an arbitrary *N*, we propose an upper bound and a lower bound of the channel capacity to draw the following conclusion.

**Proposition** **1.** *For any fixed total transmit power P and number of encoders N, a linear growth of total fronthaul capacity $C_{B}$ results in a logarithmical growing of the channel capacity as C can be bounded as*

(43) $$C\leq \frac{1}{2}\log_{2}\left(1+P\right)+\frac{1}{2}\log_{2}\left(1+2\ln2\cdot C_{B}\right),$$

*and*

(44) $$C>\frac{1}{2}\log_{2}\left(1+{\left(2\ln2\cdot PC_{B}\right)}^{2/3}\right).$$

*for $C_{B}\leq \left(N-1\right)C_{\mathrm{full}}$.*

**Proof.**  *(1) Upper bound.* By considering L∈[1:N] as a random variable with distribution $\underline{\beta }$, the capacity (38) can be expressed as

(45) $$C=\frac{1}{2}\log_{2}\left(1+\mu_{L}P\right),$$

where $\mu_{L}≜E\left[L\right]$. By applying Jensen’s inequality, the corresponding fronthaul capacity $C_{B}$ in (39) can be lower bounded as

(46) $$\begin{array}{ccc} C_{B} & \;\geq & \;\left(N-1\right)C-\frac{N-1}{2}\log_{2}\left(\frac{\sum_{m=1}^{N-1}\left(1+\sum_{l=1}^{m}l\beta_{l}P\right)}{N-1}\right)\\ & \;= & \;\left(N-1\right)C-\frac{N-1}{2}\log_{2}\left(1+\frac{\sum_{l=1}^{N-1}\left(N-l\right)l\beta_{l}P}{N-1}\right)\\ & \;\overset{\left(a\right)}{\geq } & \;\left(N-1\right)C-\frac{N-1}{2}\log_{2}\left(1+\frac{\mu_{L}\left(N-\mu_{L}\right)P}{N-1}\right)\\ & \;= & \;\frac{N-1}{2}\log_{2}\left(\frac{1+\mu_{L}P}{1+\frac{\mu_{L}\left(N-\mu_{L}\right)P}{N-1}}\right)\\ & \;\overset{\left(b\right)}{\geq } & \;\frac{\left(\mu_{L}-1\right)\mu_{L}P}{2\ln2\cdot (1+\mu_{L}P)} \end{array}$$

where (a) follows $\mu_{L}^{2}\leq E\left[L^{2}\right]$ and (b) follows $\ln x\geq 1-\frac{1}{x}$ for x>0. Since $\mu_{L}\geq 1$, we can have $C_{B}\geq \frac{\left(\mu_{L}-1\right)\mu_{L}P}{2\ln2\cdot (\mu_{L}+\mu_{L}P)}=\frac{(\mu_{L}-1)P}{2\ln2\cdot (1+P)}$ that results in $\mu_{L}\leq 1+2\ln2\cdot \left(\frac{1+P}{P}\right)C_{B}$ and thus (43).

*(2) Lower bound.* Consider time-sharing of the rates given by only using one cooperating mode. Hence, the channel capacity should be larger than or equal to an achievable rate *R* as

(47) $$C\geq R≜\frac{1}{2}\log_{2}\left(1+kP\right),$$

where *k* is the number of the activated encoders corresponding to the required total fronthaul capacity

(48) $$C_{B}=\left(k-1\right)R$$

that achieves *R*. By applying $\ln x\leq \frac{x-1}{\sqrt{x}}$ for x≥1, see, e.g., ([19], Section 3.6.15), we have

(49) $$R\leq \frac{1}{2\ln2}\cdot \frac{kP}{\sqrt{1+kP}}$$

that gives an upper bound of $C_{B}$ as

(50) $$\begin{array}{ccc} C_{B} & \leq & \frac{(k-1)kP}{2\ln2\sqrt{1+kP}}\\ \; & < & \frac{(k-1)kP}{2\ln2\sqrt{kP}}\\ \; & < & \frac{k\sqrt{kP}}{2\ln2}. \end{array}$$

Therefore, we can have $k^{3}>{\left(2\ln2\cdot C_{B}\right)}^{2}/P$ that directly gives (44). □

**Figure 4:**
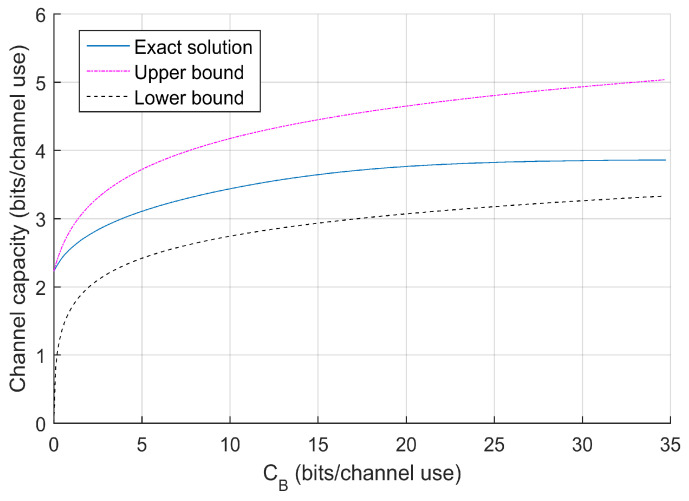
Upper and lower bounds on $C(C_{B})$ at P=21 for 10-encoder Gaussian setting.

The upper bound (43) and lower bound (44) thus indicate the logarithmical behavior of *C* in $C_{B}$. Figure 4 gives an illustration of these two bounds for 10-encoder Gaussian setting where total transmit power is set at P=21.

The exact capacity solution derived shortly is plotted as well as a comparison, showing that the bounds describes the capacity behavior.

### 4.4. Compound Mode and Exact Solution

In what follows, we perform evaluation of the channel capacity given in Theorem 5 by defining a *compound mode* 〈j,k〉 as a collection of all consecutive *cooperating modes* between and including *modes* j,k∈[1:N] with j≤k. A *compound mode* 〈j,k〉 is referred to as active if all ${\left\{\beta_{l}\right\}}_{l=j}^{k}$ are nonzero and the other elements in $\underline{\beta }$ are zeros. Note that using a single mode is a special case of compound mode. By denoting

(51) $$b\left(j\right)≜\frac{1}{j(2+(j+1)P)},$$

we have the following results.

**Corollary** **2.** *For an N-encoder Gaussian setting where $C_{B}\leq \left(N-1\right)C_{\mathrm{full}}$ with a fixed transmit power P, if there is a compound mode 〈j,k〉 such that UB≥LB, where*

(52) $$UB≜\left\{\begin{array}{ccc} \frac{1}{2(k-1)} & \mathrm{if} & j=1\\ \min\{\frac{1}{2(k-1)},b\left(j-1\right)\} & \mathrm{if} & j>1, \end{array}\right.$$

(53) $$LB≜\left\{\begin{array}{ccc} \max\{\frac{1}{2k},b\left(j\right)\} & \mathrm{if} & k<N\\ b(j) & \mathrm{if} & k=N, \end{array}\right.$$

*the channel capacity corresponding to the slope λ∈[LB,UB] is **achieved** and **only achieved** by using that compound mode which gives*

(54) $$\begin{array}{c} C\left(\lambda \right)=\frac{1}{2}\log_{2}\left(\frac{k(1-(k-1)\lambda )(1+jP)}{j(1-(j-1)\lambda )}\right), \end{array}$$

*and*

(55) $$\begin{array}{c} C_{B}\left(\lambda \right)=\frac{1}{2}\log_{2}\left(\frac{{\left(2\lambda \right)}^{j-k}{\left(1+jP\right)}^{j-1}}{\prod_{l=j}^{k-1}\frac{l(l+1)}{2}}\cdot \frac{{\left(k\left(1-\left(k-1\right)\lambda \right)\right)}^{k-1}}{{\left(j\left(1-\left(j-1\right)\lambda \right)\right)}^{j-1}}\right). \end{array}$$

The proof is given in Appendix A.4.

**Remark** **4.** *The proof in Appendix A.4 shows that if compound mode 〈j,k〉 achieves the capacity and k≥j+2, the modes in [j+1:k−1] should be assigned with same power amount as the optimal setting.*

**Remark** **5.** *By rewriting (55) and comparing it to (54), we can also represent $C_{B}$ in terms of C as*

(56) $$\;C_{B}\left(\lambda \right)=\left(j-1\right)C\left(\lambda \right)+\frac{1}{2}\log_{2}\left(\frac{{\left[\lambda^{-1}k\left(1-\left(k-1\right)\lambda \right)\right]}^{k-j}}{\prod_{l=j}^{k-1}l\left(l+1\right)}\right).$$

*for a certain slope λ∈[LB,UB].*

### 4.5. Modes Selection for Capacity Achieving

The results in Corollary 2 state how the capacity is achieved and expressed over a certain λ range. To further elaborate how to exactly use cooperating modes from no cooperation to full cooperation, a procedure efficiently activating modes is developed based on applying the following result, where an identification of valid compound modes that are the ones resulting in capacity is provided in terms of using a power penalty.

**Corollary** **3.** *For any fixed P, a compound mode 〈j,k〉 achieves the capacity if and only if*

(57) $$\begin{array}{c} \frac{2(k-j-1)}{j(j+1)}<P\leq \frac{2(k-j+1)}{j\left(j-1\right)\left\lceil 1-\frac{k}{N}\right\rceil }, \end{array}$$

*where ⌈·⌉ is the ceiling function. If (57) is satisfied, compound modes $\langle j^{\prime },k^{\prime }\rangle$ with $j^{\prime }>j$ and $k^{\prime }<k$ do not achieve the capacity.*

The proof is given in Appendix A.5. The power condition (57) indicates that a compound mode needs certain transmit power to be supported to be optimal. On the other hand, some compound modes can never be optimal if the transmit power is too large.

Now, note that *C* is monotonically increasing in $C_{B}$ owing to nonnegative slope λ and monotonically decreasing in λ according to (54) when *j* and *k* are fixed. It shows that to achieve the capacity, *compound modes* should be activated in a way such that the corresponding slope range varies from large to small as $C_{B}$ increases. Therefore, based on the results in Corollary 3, an algorithm is resulted for computing *C* and $C_{B}$ over $C_{B}\in \left[0,\left(N-1\right)C_{\mathrm{full}}\right]$ by activating valid compound modes sequentially.

Algorithm 1 represents the cooperating strategy among encoders. It reveals that cooperating modes should be activated one-by-one to form new compound modes with the increase of $C_{B}$. At certain point of the growth of $C_{B}$, the first mode dies, i.e., deactivated owing to the limited *P* or *N*. With the further increasing of $C_{B}$, lower modes die in a one-by-one fashion till the full cooperation is obtained.

**Figure 5:**
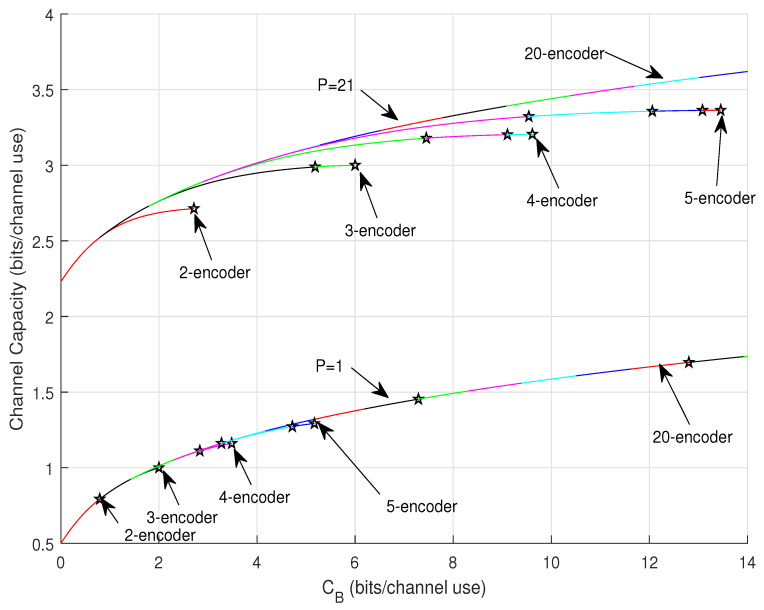
$C(C_{B})$ at P=1 and P=21 for different *N*-encoder settings.

Figure 5 plots the results for P=1 and P=21 by applying Algorithm 1, $C(C_{B})$ over the full range of $C_{B}\in \left[0,\left(N-1\right)C_{\mathrm{full}}\right]$. Different number of the available encoders are considered. In the plot, each color segment represents the corresponding activated compound mode. In addition, the pentagram markers label the points where a lower mode has to be deactivated (dead) because of the power penalty (57) or because all *N* encoders are all used up, namely operations in line 13 and line 9 of the algorithm, respectively. It is shown that for low SNR, i.e., P=1, the modes die fast due to the small power. On the other hand, for large SNR, i.e., P=21, the larger available encoder number the slower the modes die such that higher capacity can be achieved (consider curves of using 2-encoder, 3-encoder, 4-encoder, and 5-encoder).

**Algorithm 1** Compute *C* and $C_{B}$ from no cooperation to full cooperation**Initialize:**j←1 and k←1
**Ensure:**
1≤j≤k≤N
1:**while**j<N**do**2: **if** Power condition (57) is satisfied **then**3:  λ←[LB,UB]4:  Compute C(λ) and $C_{B}\left(\lambda \right)$ by (54) and (55)5:  **if** k<N **then**6:   k←k+17:  **else**8:   k←N9:   j←j+110:  **end if**11: **else**12:  k←k−113:  j←j+114: **end if**15:**end while**

**Proposition** **2.** *As probably the most natural strategy, the way of applying modes in the lower bound proof of Proposition 1, i.e., time-sharing full cooperation of small number of encoders, is not optimal in general. However, it is sub-optimal when SNR is small as the compound modes that achieve capacity reduce to single modes.*

**Figure 6:**
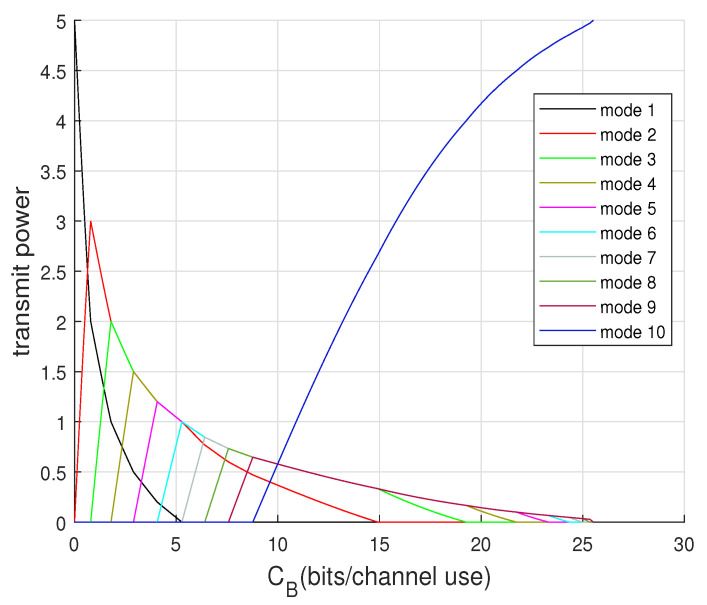
Optimal power allocation for modes distribution for 10-encoder Gaussian setting at P=5.

**Figure 7:**
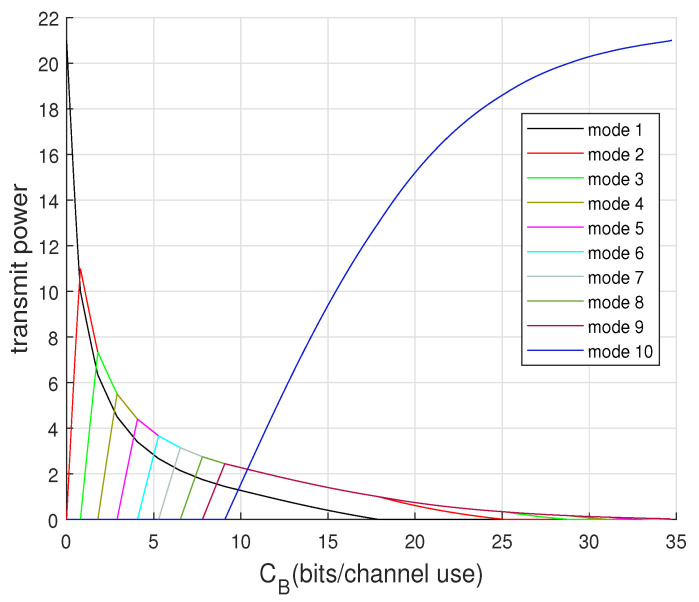
Optimal power allocation for modes distribution for 10-encoder Gaussian setting at P=21.

To visualize each mode evolution from no beamforming to total beamforming, we can illustrate the power allocation for each cooperating mode as $C_{B}$ increases. By incorporating calculations of $\underline{\beta }$ (given in Appendix A.4) and (37) into Algorithm 1, Figure 6 and Figure 7 show the modes’ power evolution of the 10-encoder setting for P=5 and P=21, respectively. It is shown that the first mode dies faster when *P* is relatively small. They also interestingly show that once $C_{B}$ is large enough to approach the total beamforming, the last mode dominates as other modes all vanish.

**Figure 8:**
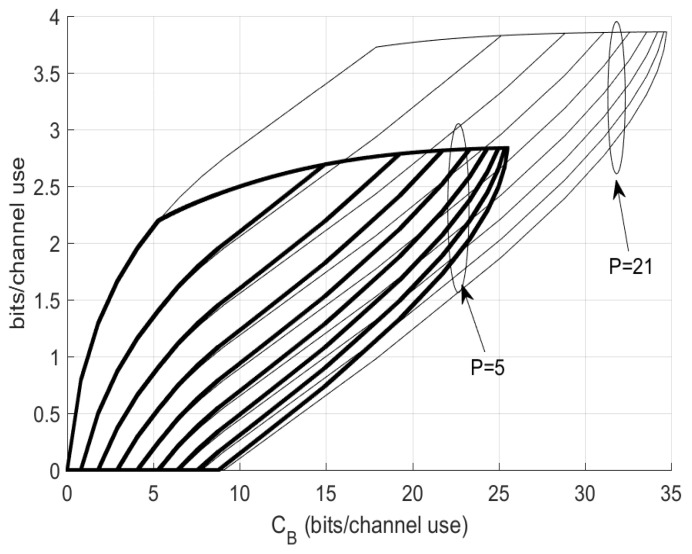
Optimal distribution of $C_{B}$ at P=5 and P=21 for 10-encoder Gaussian setting.

Moreover, we can also elaborate the cooperating of encoders in terms of showing optimal distribution of $C_{B}$ over fronthaul links. Figure 8 illustrates the distribution of the 10-encoder setting where the bolder curves are for P=5 while the lighter curves are for P=21. In each case, the fronthaul capacity curves for $C_{m\;m+1}$ for m=1 to m=9 are located from left to right in the plot. This result further demonstrates the asymmetric water-pouring assignment of $C_{B}$ over fronthaul links, see Remark 3.

### 4.6. 〈1,k〉 Mode and Capacity Regimes

Consider the case where 〈1,k〉 mode achieves the capacity for k≤N. In this case, the growth rate of $C(C_{B})$ is independent of *P* and *N*, see the expression of $C_{B}$ in (56) with j=1. Therefore, we call that the system works in a fronthaul-capacity-limited regime when a 〈1,k〉 mode is used. The reason why we are interested in the fronthaul-capacity-limited regime is that $C(C_{B})$ achieves the fast growth rate regardless of *P* and *N*. As a further increase of $C_{B}$, the first mode dies due to either limited *P* or limited *N*. We then call the system works in a power-limited regime or encoder-limited regime, respectively. When the system is in either power-limited regime or encoder-limited regime, $C(C_{B})$ growth is slowed down compared to when the system works in the fronthaul-capacity-limited regime. This is due to the discontinuities of the slope λ, see the derived optimal upper and lower bounds of λ. The following result shows how to determine which regime the system works in for given $C_{B}$, *P*, and *N*.

**Proposition** **3.** *For given P and N, if*

(58) P≤N−2,

*the capacity growth is limited by P and the system works in a fronthaul-capacity-limited regime if*

(59) $$C_{B}⪅\frac{1}{2}\log_{2}(\frac{{\left(\left(1+\tilde{P}\right)\left(2+\tilde{P}\right)\right)}^{\tilde{P}}}{\tilde{P}!\left(\tilde{P}+1\right)!}),$$

*where $\tilde{P}≜\left\lceil P\right\rceil$. Otherwise it works in a power-limited regime.*

*On the other hand, if P>N−2, the capacity growth is limited by N and the system works in a fronthaul-capacity-limited regime if*

(60) $$C_{B}\leq \frac{1}{2}\log_{2}(\frac{{\left(N\left(3+2P-N\right)\right)}^{N-1}}{(N-1)!N!}).$$

*Otherwise it works in a encoder-number-limited regime.*

**Proof.**  Modes dying hampers the growth of *C* in $C_{B}$. Consider that the first mode of the compound mode 〈1,k〉 dies because of constrained *P* not *N*. In this case, *P* must satisfy (58) which is given by the lower bound of (57). Consequently, at the moment after the first mode dies, i.e., compound mode 〈2,k〉 is active, we have j=2 and k≈⌈P⌉+1 given by taking the upper bound of (57). This j,k setting results in $\lambda =\frac{1}{2+2P}$ so that (59) is obtained by evaluating (55).

Similarly, considering the compound mode 〈1,N〉 can be supported by *P*, the first mode dies because that no new encoders can be used. At the moment of first mode dying, i.e., compound mode 〈1,N〉 is still active, we thus have j=1 and k=N, which also result in $\lambda =\frac{1}{2+2P}$. Hence, (60) is resulted. □

**Figure 9:**
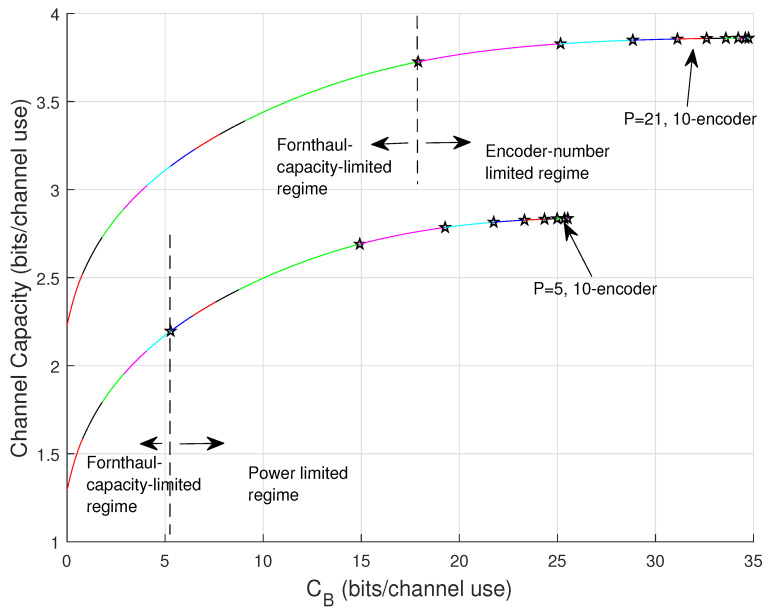
$C(C_{B})$ at P=5 and P=21 for 10-encoder Gaussian setting.

Figure 9 plots $C(C_{B})$ of 10-encoder setting for P=5 and P=21, respectively, where the regime separations are indicated at the first mode dies for both powers. It is illustrated that in the fronthaul-capacity-limited regime, *C* has the highest growth rate no matter what its initial value is (point-to-point communication). In the next subsection, we focus on a system working at the fronthaul-capacity-limited regime.

## 5. Infinitely Many Encoders

Consider designing a system in practice when $C_{B}$ and *P* are critical resources while available encoders could be many, for instance, the radio stripe system. Based on the previous study, we should always try to let the system work in its fronthaul-capacity-limited regime where the fronthaul capacity is maximally utilized. Hence, we are motivated to determine the number of encoders that are required to be activated for a given $C_{B}$ by considering infinitely many of them are available when the system is purely fronthaul constrained.

To directly solve *k*, the highest active mode that is the number of required encoders, from (55) or (56) is not trivial. To achieve an accurate approximate result, we first need the following lemma, of which the proof follows the outline in [20] and is given in Appendix A.6.

**Lemma** **2.** *The non-principle branch of Lambert W function $W_{-1}\left(\cdot \right)$ defined in the interval $\left[-e^{-1},0\right)$, see [21], can be bounded as follows*

(61) $$W_{-1}\left(-e^{-(x+1)}\right)\geq -x-\sqrt[{3}]{2x}-1$$

*for x≥0.*

**Figure A3:**
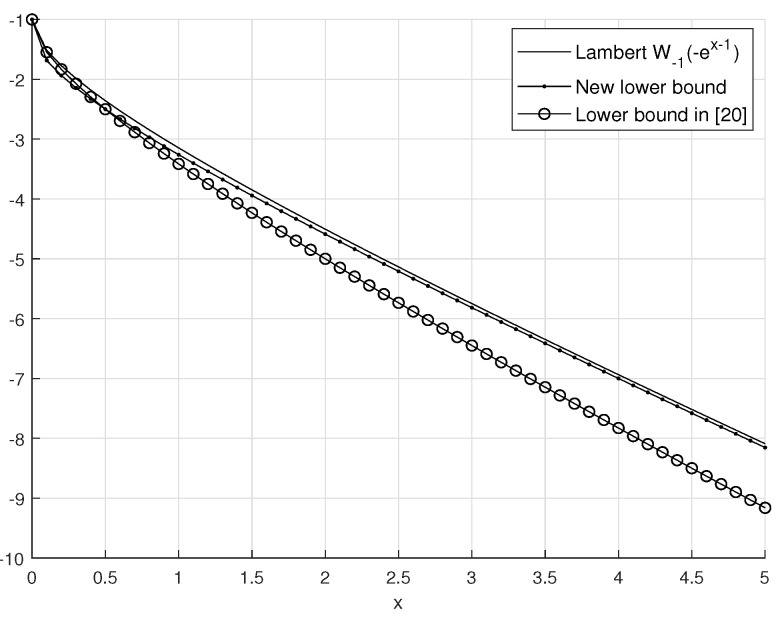
The bounds on the Lambert function $W_{-1}\left(-e^{-(x+1)}\right)$.

**Remark** **6.** *Figure A3 shows that for ∀x≥0.5, the lower bound (61) is much tighter than $W_{-1}\left(-e^{-(x+1)}\right)\geq -x-\sqrt{2x}-1$ given in [20], which is the tightest bound of $W_{-1}\left(\cdot \right)$ reported in the literature so far, to our best knowledge.*

**Proposition** **4.** *When the system works in the fornthaul limited regime, the number of required encoders $\tilde{k}$ is quasi-linear to the available fronthaul capacity as*

(62) $$\tilde{k}=\left\lfloor C_{B}+{\left(2C_{B}+1\right)}^{1/3}+1.5\right\rfloor ,$$

*where $C_{B}$ in nats per channel use and ⌊·⌋ is the floor function.*

**Proof.**  As the system works in the fronthaul-limited regime, compound mode 〈1,k〉 exists. Thus, according to (56),

(63) $$C_{B}=\frac{1}{2}\ln\left(\frac{{\left[\lambda^{-1}k\left(1-\left(k-1\right)\lambda \right)\right]}^{k-1}}{\prod_{l=1}^{k-1}l\left(l+1\right)}\right)$$

in nats. To upper bound *k*, we lower bound $C_{B}$ by taking $\lambda =\frac{1}{2(k-1)}$, see the bound (52), which gives

(64) $$\begin{array}{ccc} C_{B} & \geq & \frac{1}{2}\ln\left(\frac{k^{k-2}{\left(k-1\right)}^{k-1}}{{\left(\left(k-1\right)!\right)}^{2}}\right)\\ & \overset{\left(a\right)}{\geq } & \frac{1}{2}\ln\left(\frac{e^{2(k-1)}k^{k-2}}{e^{2}{\left(k-1\right)}^{k}}\right)\\ & = & k-2-\ln k+\frac{k}{2}\ln\left(\frac{k}{k-1}\right)\\ & \overset{\left(b\right)}{\geq } & k-\ln k-1.5 \end{array}$$

where (a) follows by applying $\left(k-1\right)!\leq e{\left(k-1\right)}^{k-\frac{1}{2}}e^{-k+1}$ derived based on ([22], 6.1.38), and (b) follows by taking the fact that ${\left(\frac{k}{k-1}\right)}^{k}$ is monotonically decreasing in *k* and goes to *e* as k→∞. Therefore, we have

(65) $$-\exp\left(C_{B}+1.5\right)>-k\exp\left(-k\right).$$

Now, solving *k* and applying (61) give

(66) $$\begin{array}{ccc} k & \leq & -W_{-1}\left(-\exp(-\left(C_{B}+1.5\right))\right)\\ \; & \leq & C_{B}+\sqrt[{3}]{2C_{B}+1}+1.5. \end{array}$$

Finally, since the number of encoders is a integer, (62) is resulted. □

**Figure 10:**
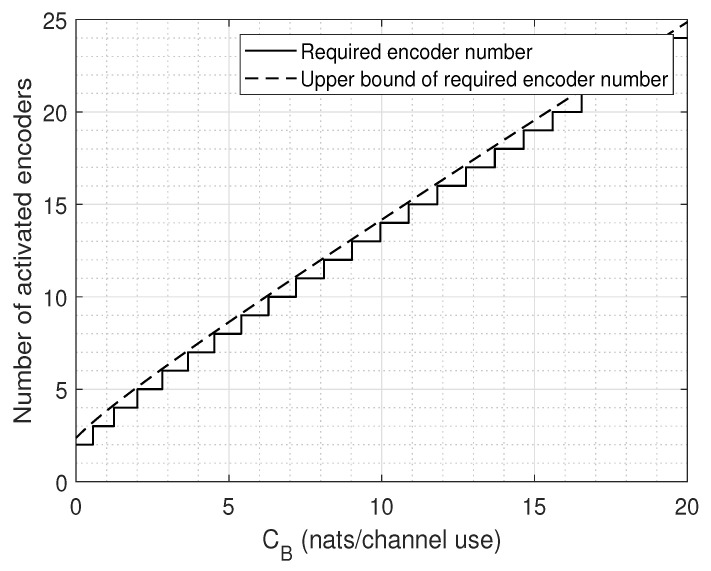
Number of required encoders in relation to $C_{B}$ for fronthaul resource maximum usage.

In Figure 10, the bound of *k* given by (66) and the actual number encoders required to be activated are plotted as a function of $C_{B}$. It is revealed that the derived result is accurate enough.

## 6. Concluding Remarks

In this paper, the downlink of a cell-free mMIMO in which multiple APs connected in a linear fronthaul topology serve as a single receiver was studied to reveal relations between the three fundamental network resources, namely the total fronthaul capacity $C_{B}$, the total transmit number *P*, and the number of available APs *N*. Specifically, we focused on partial distributed beamforming where the total available fronthaul capacity is not enough to support full cooperation between all APs, i.e., beamforming. By formulating the problem as a MAC channel with multiple encoders linked in a feed-and-forward setting, we derived the channel capacity as a function of the total fronthaul capacity for both discrete and Gaussian channels. The derivation was started by considering two encoders and then we extended the analysis multiple encoders. It was demonstrated that capacity is achieved by multi-layer superposition coding from which the concept of cooperating mode was developed for the Gaussian channel. This cooperating mode technique leads to optimal cooperation among encoders. Bounds on the capacity for *N*-encoder setting demonstrated that this channel capacity grows logarithmically in $C_{B}$ for a fixed *P*. The exact capacity solution shows that the capacity is achieved if and only if by certain compound modes are used. An algorithm was derived for computing which compound modes should be activated as as function of $C_{B}$, which grows from zero to the value obtaining full beamforming. We demonstrated that $C_{B}$ should be water-poured over the fronthaul links to obtain optimality. Finally, by considering the case where infinitely many encoders are available, we showed that the number of required encoders is quasi-linear to the available total fronthaul capacity when the system is purely constrained by fronthaul resources.

Future directions include extending the results to channels with links which do not have unit gain as is the case here, and considering multiple receivers. Another interesting direction would be the equivalent uplink case.

## Figures and Tables

**Figure A1:**
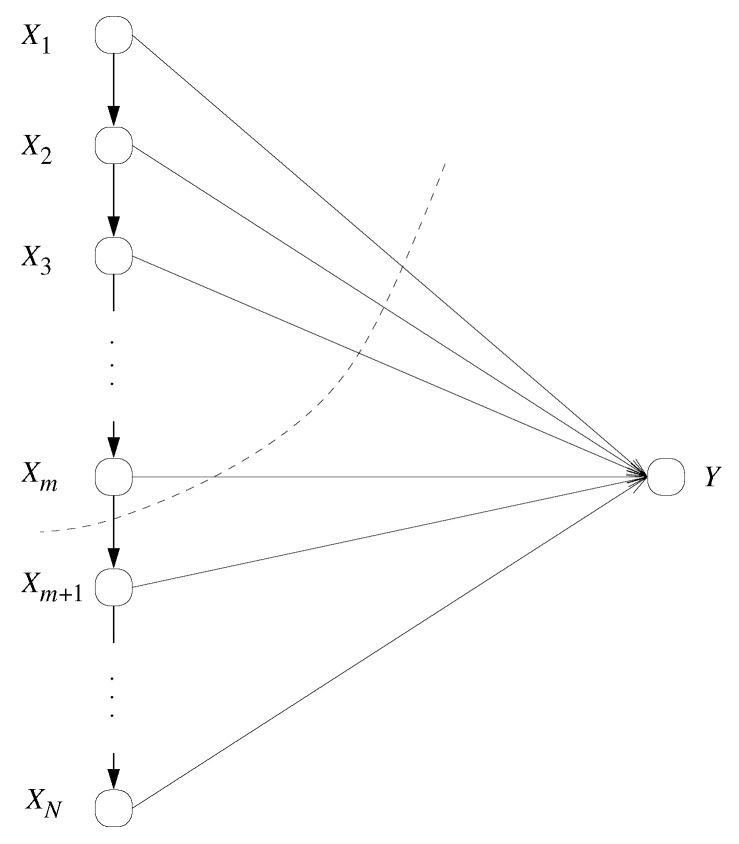
Simplified illustration of the MAC for the *N*-encoder setting.

**Figure A2:**
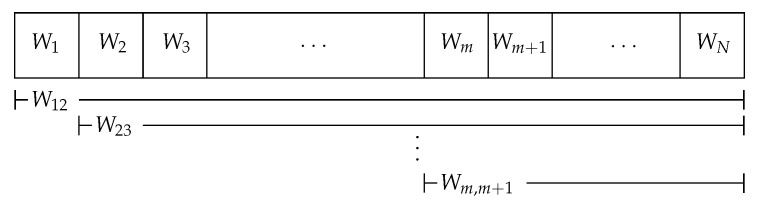
*N*-layer superposition coding message structure.

## Appendix A. Proofs and Derivations

### Appendix A.1. Proof of Theorem 1

(i) *Converse.* Consider Fano’s inequality $H\left(W|Y^{n}\right)\leq 1+P_{e}^{\left(n\right)}\log_{2}M=F$ for $F\overset{\Delta }{=}1+P_{e}^{\left(n\right)}\log_{2}M$. Since $W\to \left(X_{1}^{n},X_{2}^{n}\right)\to Y^{n}$ forms a Markov chain, we have that

(A1) $$\begin{array}{ccc} \log_{2}M=H\left(W\right) & = & I\left(W;Y^{n}\right)+H\left(W|Y^{n}\right)\leq I\left(W,X_{1}^{n},X_{2}^{n};Y^{n}\right)+F\\ \; & \leq & I\left(X_{1}^{n},X_{2}^{n};Y^{n}\right)+F\leq \sum_{i=1}^{n}I\left(X_{1i},X_{2i};Y_{i}\right)+F\\ \; & \leq & nI\left(X_{1},X_{2};Y|Q\right)+F\leq nI\left(X_{1},X_{2};Y\right)+F. \end{array}$$

Moreover, from the Markovity of $\left(W,W_{12}\right)\to \left(X_{1}^{n},X_{2}^{n}\right)\to Y^{n}$, we obtain that

(A2) $$\begin{array}{ccc} \log_{2}M=H\left(W\right) & = & I\left(W;Y^{n}\right)+H\left(W|Y^{n}\right)\leq I\left(W,W_{12};Y^{n}\right)+F\\ \; & = & I\left(W_{12};Y^{n}\right)+I\left(W;Y^{n}|W_{12}\right)+F\leq \log_{2}M_{12}+I\left(X_{1}^{n};Y^{n}|X_{2}^{n}\right)+F\\ \; & \leq & nC_{12}+I\left(X_{1}^{n};Y^{n}|X_{2}^{n}\right)+F\leq nC_{12}+\sum_{i=1}^{n}I\left(X_{1i};Y_{i}|X_{2i}\right)+F\\ \; & \leq & nC_{12}+nI\left(X_{1};Y|X_{2},Q\right)+F\leq nC_{12}+nI\left(X_{1};Y|X_{2}\right)+F. \end{array}$$

In the above derivations, the random variable *Q* is uniformly distributed on [1:n] and $\mathrm{Pr}\left\{X_{1}=x_{1},X_{2}=x_{2}\right\}=\frac{1}{N}\sum_{i=1}^{N}\mathrm{Pr}\left\{X_{1i}=x_{1},X_{2i}=x_{2}\right\}$ for $x_{1}\in X_{1},x_{2}\in X_{2}$. If now both δ→0 and n→∞ we obtain that

(A3) $$R\leq \min\{I\left(X_{1},X_{2};Y\right),I\left(X_{1};Y|X_{2}\right)+C_{12}\},$$

for some distribution $p\left(x_{1},x_{2},y\right)=p\left(x_{1},x_{2}\right)p\left(y|x_{1},x_{2}\right)$, for all achievable rate *R*. This concludes the converse for the discrete memoryless two-encoder case.

(ii) *Achievability.* We prove that if the message rate $\left(1/n\right)\log_{2}M<C_{B}\left(C_{12}\right)$ for a given fronthaul capacity $C_{12}$, the message error probability $P_{e}^{\left(n\right)}$ approaches zero if the codeword length *n* increases. Our coding method is based on superposition.

*Codebook Generation*: First fix a joint probability distribution $\left\{p\left(x_{1},x_{2}\right),x_{1}\in X_{1},x_{2}\in X_{2}\right\}$. This distribution determines $p_{X_{2}}\left(x_{2}\right)=\sum_{x_{1}\in X_{1}}p\left(x_{1},x_{2}\right)$ and $p_{X_{1}|X_{2}}\left(x_{1}|x_{2}\right)=p\left(x_{1},x_{2}\right)/p_{X_{2}}\left(x_{2}\right)$ for $x_{2}$ with $p_{X_{2}}\left(x_{2}\right)>0$. Now generate at random $M_{2}$ i.i.d. sequences $x_{2}^{n}\in {X_{2}}^{n}$ of length *n*, each drawn according to $\mathrm{Pr}\left\{X_{2}^{n}=x_{2}^{n}\right\}=\prod_{i=1}^{n}p_{X_{2}}\left(x_{2i}\right)$ and index these sequences as $x_{2}^{n}\left(w_{2}\right)$ as an inner code, where $w_{2}\in \left[1:M_{2}\right]$. Then, for each such $x_{2}^{n}\left(w_{2}\right)$, generate $M_{1}$ sequences $x_{1}^{n}\left(w_{1},w_{2}\right)$ drawn according to $\mathrm{Pr}\left\{X_{1}^{n}=x_{1}^{n}|X_{2}^{n}=x_{2}^{n}\left(w_{2}\right)\right\}=\prod_{i=1}^{n}p_{X_{1}|X_{2}}\left(x_{1i}|x_{2i}\left(w_{2}\right)\right)$ in an i.i.d. fashion as an outer code, where $w_{1}\in \left[1:M_{1}\right]$. The resulting codebook is revealed to both encoders and to the decoder.

*Encoding*: Split the message *W* that is uniformly distributed on [1:M] into $\left(W_{1},W_{2}\right)$ with $M=M_{1}\times M_{2}$, where the first part $W_{1}$, which is uniformly distributed on $\left[1:M_{1}\right]$, is transmitted by encoder 1 and the second part $W_{2}$, which is uniformly distributed on $\left[1:M_{2}\right]$ and is conveyed to encoder 2 by $W_{12}$, is transmitted by two encoders cooperatively. Hence, when $\left(W_{1},W_{2}\right)=\left(w_{1},w_{2}\right)$, encoder 2 sends $x_{2}^{n}\left(w_{2}\right)$ while encoder 1 inputs $x_{1}^{n}\left(w_{1},w_{2}\right)$ into the MAC.

*Decoding*: Let ϵ>0. Based on the observed channel output sequence $y^{n}$, the decoder finds the message pair $\left(w_{1},w_{2}\right)$ such that

(A4) $$\left(x_{1}^{n}\left(w_{1},w_{2}\right),x_{2}^{n}\left(w_{2}\right),y^{n}\right)\in A_{\epsilon }^{\left(n\right)}\left(X_{1}X_{2}Y\right),$$

where set $A_{\epsilon }^{\left(n\right)}\left(X_{1}X_{2}Y\right)$ is the set of jointly ϵ-typical sequences, see Cover and Thomas [18]. If such a pair cannot be found, or if there are more than one such pairs, an error is declared.

*Probability of Error*: Due to symmetry, the average probability of error is equivalent to the probability of error for an arbitrary message $w\in \{1,\ldots ,2^{nR}\}$. Hence, without loss of generality, we assume $W=w=(w_{1},w_{2})$. Thus, we have

(A5) $$\begin{array}{ccc} P_{e}^{\left(n\right)} & = & \mathrm{Pr}\{E^{c}\left(w_{1},w_{2}\right)\cup \underset{\left({\tilde{w}}_{1},{\tilde{w}}_{2}\right)\neq \left(w_{1},w_{2}\right)}{⋃}E\left({\tilde{w}}_{1},{\tilde{w}}_{2}\right)\}\\ \; & \leq & \mathrm{Pr}\left\{E^{c}\left(w_{1},w_{2}\right)\right\}+\sum_{\left({\tilde{w}}_{1},{\tilde{w}}_{2}\right)\neq \left(w_{1},w_{2}\right)}\mathrm{Pr}\left\{E\left({\tilde{w}}_{1},{\tilde{w}}_{2}\right)\right\}\\ \; & = & \mathrm{Pr}\left\{E^{c}\left(w_{1},w_{2}\right)\right\}+\sum_{{\tilde{w}}_{1}\neq w_{1}}\mathrm{Pr}\left\{E\left({\tilde{w}}_{1},w_{2}\right)\right\}+\sum_{\left({\tilde{w}}_{1},{\tilde{w}}_{2}\right):{\tilde{w}}_{2}\neq w_{2}}\mathrm{Pr}\left\{E\left({\tilde{w}}_{1},{\tilde{w}}_{2}\right)\right\}, \end{array}$$

where

(A6) $$E\left(w_{1},w_{2}\right)\overset{\Delta }{=}\left\{\left(x_{1}^{n}\left(w_{1},w_{2}\right),x_{2}^{n}\left(w_{2}\right),Y^{n}\right)\in A_{\epsilon }^{\left(n\right)}\left(X_{1}X_{2}Y\right)\right\}.$$

Due to the Asymptotic Equipartition Property (AEP), it can be shown that

(A7) $$\mathrm{Pr}\{E^{c}\left(w_{1},w_{2}\right)\}\leq \epsilon ,$$

for all *n* large enough. Moreover

(A8) $$\begin{array}{ccc} \sum_{{\tilde{w}}_{1}\neq w_{1}}\mathrm{Pr}\left\{E\left({\tilde{w}}_{1},w_{2}\right)\right\}\; & \leq & \left(M_{1}-1\right)\sum_{\left(x_{1}^{n},x_{2}^{n},y^{n}\right)\in A_{\epsilon }^{\left(n\right)}}P\left(x_{1}^{n}|x_{2}^{n}\right)P\left(x_{2}^{n}\right)P\left(y^{n}|x_{2}^{n}\right)\\ \; & \leq & \left(M_{1}-1\right)2^{-n(I\left(X_{1};Y|X_{2}\right)-4\epsilon )}, \end{array}$$

and

(A9) $$\begin{array}{ccc} \sum_{\left({\tilde{w}}_{1},{\tilde{w}}_{2}\right):{\tilde{w}}_{2}\neq w_{2}}\mathrm{Pr}\left\{E\left({\tilde{w}}_{1},{\tilde{w}}_{2}\right)\right\}\; & = & M_{1}\left(M_{2}-1\right)\sum_{\left(x_{1}^{n},x_{2}^{n},y^{n}\right)\in A_{\epsilon }^{\left(n\right)}}P\left(x_{1}^{n}|x_{2}^{n}\right)P\left(x_{2}^{n}\right)P\left(y^{n}\right)\\ \; & \leq & M_{1}\left(M_{2}-1\right)2^{-n(I\left(X_{1},X_{2};Y\right)-3\epsilon )}. \end{array}$$

Now as long as

(A10) $$\begin{array}{ccc} M_{1} & \leq & 2^{n(I\left(X_{1};Y|X_{2}\right)-5\epsilon )},\\ M_{1}M_{2} & \leq & 2^{n(I\left(X_{1},X_{2};Y\right)-4\epsilon )}, \end{array}$$

$P_{e}^{\left(n\right)}\leq 2\epsilon$ for all *n* large enough. Therefore we take

(A11) $$\begin{array}{ccc} \log_{2}M_{2} & = & \min\{n\left(I\left(X_{2};Y\right)+\epsilon \right),nC_{12}\},\\ \log_{2}M_{1} & = & n(I\left(X_{1};Y|X_{2}\right)-5\epsilon ), \end{array}$$

then both (9) and (A10) are satisfied. Note that this implies that

(A12) $$\begin{array}{ccc} \log_{2}M_{1}M_{2} & = & \min\{n\left(I\left(X_{1};Y|X_{2}\right)-5\epsilon \right)+nC_{12},n\left(I\left(X_{1},X_{2};Y\right)-4\epsilon \right)\}. \end{array}$$

If we now let ϵ→0, the achievability part of the Theorem 1 is thus established.

### Appendix A.2. Proof of Theorem 3

The proof is a generalization of the proof of the two-encoder settings. Consider a simplified block diagram of the *N*-encoder setting as shown in Figure A1. Now, consider a cut of the fronthaul link between $X_{m}$ and $X_{m+1}$ for any given m∈[1:N−1] such that the nodes in the network are separated in two sets of $\left\{{\underline{X}}_{1}^{m}\right\}$ and $\left\{{\underline{X}}_{m+1}^{N},Y\right\}$.

(i) *Converse*. Consider the Markovity of $W\to (X_{1}^{n},X_{2}^{n},$ $\ldots ,X_{N}^{n})\to Y^{n}$. By applying Fano’s inequality, we first have

(A13) $$\begin{array}{ccc} \log_{2}M & \leq & I\left(W,W_{12},\ldots ,W_{N-1,N};Y^{n}\right)+H\left(W|Y^{n}\right)\\ \; & \leq & I(X_{1}^{n},X_{2}^{n},\ldots ,X_{N}^{n};Y^{n})+F\\ \; & \leq & \sum_{i=1}^{n}I\left(X_{1i},X_{2i},\ldots ,X_{N_{i}};Y_{i}\right)+F\\ \; & \leq & nI({\underline{X}}_{1}^{N};Y)+F. \end{array}$$

Then, considering the cut between $X_{m}$ and $X_{m+1}$, we have that

(A14) $$\begin{array}{ccc} \log_{2}M & = & I\left(W,W_{12},\ldots ,W_{N-1,N};Y^{n}\right)+H\left(W|Y^{n}\right)\\ \; & \leq & I\left(W_{m,m+1};Y^{n}\right)+I\left(W,W_{12},\ldots ,W_{m-1,m};Y^{n}|W_{m,m+1},\ldots ,W_{N-1,N}\right)+F\\ \; & \leq & \log_{2}M_{m,m+1}+I\left(X_{1}^{n},X_{2}^{n},\ldots ,X_{m}^{n};Y^{n}|X_{m+1}^{n},X_{N}^{n}\right)+F\\ \; & \leq & nC_{m,m+1}+\sum_{i=1}^{n}I\left(X_{1i},X_{2i},\ldots ,X_{m_{i}};Y_{i}|X_{m+1,i},\ldots ,X_{N,i}\right)+F\\ \; & \leq & nC_{m,m+1}+nI\left({\underline{X}}_{1}^{m};Y|{\underline{X}}_{m+1}^{N}\right)+F. \end{array}$$

Note that the above result is valid for any *m* in [1:N−1]. Thus, by letting n→∞ the converse follows.

(ii) *Achievability*. First consider the message *W* that can be represented by *N* independent messages as $W={\left\{W_{i}\right\}}_{i=1}^{N}$, where each $W_{i}$ is uniformly distributed on $\left[1:M_{i}\right]$ with $\prod_{N}^{i=1}=M$. Then, given by the linear topology of encoders, we distribute ${\left\{W_{i}\right\}}_{i=1}^{N}$ into the network in the manner illustrated by Figure A2, i.e., for the link between any $X_{m}$ and $X_{m+1}$, the fronthaul message $W_{m,m+1}$ conveys corresponding messages ${\left\{W_{i}\right\}}_{i=m}^{N}$. Therefore, for a fixed distribution $p(x_{1},x_{2},\ldots ,x_{N})$ and corresponding marginals, we can first generate $M_{N}$ i.i.d. *n*-sequences $x_{N}^{n}\left(w_{N}\right)$ with $w_{N}\in \left[1,M_{N}\right]$ according to $\mathrm{Pr}\left(X_{N}^{n}=x_{n}^{N}\right)=\prod_{i=1}^{n}p_{X_{n}}\left(x_{ni}\right)$ and then for each $x_{N}^{n}\left(w_{N}\right)$ generate $M_{N-1}$ i.i.d. *n*-sequences $x_{N-1}^{n}\left(w_{N-1},w_{N}\right)$ with $w_{N-1}\in \left[1,M_{N-1}\right]$ according to $\mathrm{Pr}\left(X_{N-1}^{n}=x_{N-1}^{n}|X_{N}^{n}=x_{n}^{N}\left(w_{N}\right)\right)=\prod_{i=1}^{n}p_{X_{n-1}|X_{n}}\left(x_{n-1\;i}|x_{ni}\left(w_{N}\right)\right)$ and so on. In this way, an *N*-layer superposition codebook is generated and revealed at both encoders and decoder.

Thus, for sending a message *w*, encoders transmit sequences ${\left\{x_{m}^{n}\left(w_{m},w_{m+1},\ldots ,w_{N}\right)\right\}}_{m=1}^{N}$ over the MAC channel. At the decoder, a unique message tuple $\left(w_{1},w_{2},\ldots ,w_{N}\right)$ is found by using simultaneous typicality decoding as performed for the two-encoder case. By taking the similar probability of error analysis, it gives that, as long as

(A15) $$\begin{array}{ccc} M_{1} & \leq & 2^{n(I\left(X_{1};Y|{\underline{X}}_{2}^{N}\right)-5\epsilon )},\\ M_{1}M_{2} & \leq & 2^{n(I\left(X_{1},X_{2};Y|{\underline{X}}_{3}^{N}\right))-5\epsilon )},\\ \; & \vdots \\ \prod_{m=1}^{N}M_{m} & \leq & 2^{n(I\left({\underline{X}}_{1}^{N};Y\right)-4\epsilon )}, \end{array}$$

we can have $P_{e}^{\left(n\right)}\leq 2\epsilon$ for all sufficiently large *n* and any ϵ>0. By further considering $M_{N}\leq 2^{nC_{N-1,N}}$, $M_{N-1}M_{N}\leq 2^{nC_{N-2,N-1}}$, *…*, and $\prod_{m=2}^{N}\leq 2^{nC_{12}}$, we can subsequently take

(A16) $$\begin{array}{ccc} \log_{2}M_{N} & = & \min\{n\left(I\left(X_{N};Y\right)+\epsilon \right),nC_{N-1,N}\}\\ \log_{2}M_{N-1} & = & \min\{nI\left(X_{N-1};Y|X_{N}\right),nC_{N-2,N-1}-\log_{2}M_{N}\}\\ \; & \vdots \\ \log_{2}M_{1} & = & n(I\left(X_{1};Y|{\underline{X}}_{2}^{N}\right)-5\epsilon ). \end{array}$$

Finally, observe that (again subsequently)

(A17) $$\begin{array}{ccc} \log_{2}M_{N} & = & \min\{n\left(I\left(X_{N};Y\right)+\epsilon \right),nC_{N-1,N}\}\\ \log_{2}M_{N-1}M_{N} & = & \min\{I\left(X_{N-1},X_{N};Y\right)+\epsilon ,\\ \; & \; & I\left(X_{N-1};Y|X_{N}\right)+nC_{N-1,N},nC_{N-2,N-1}\}\\ \; & \vdots \\ \log_{2}M & = & \log_{2}\prod_{m=1}^{N}M_{m}\\ \; & = & \min\{n(I\left({\underline{X}}_{1}^{N};Y\right)-4\epsilon ),\\ \; & \; & {\left\{I\left({\underline{X}}_{1}^{m};Y|{\underline{X}}_{m+1}^{N}\right)+C_{m,m+1}\right\}}_{m=1}^{N-1}\}, \end{array}$$

which establishes the achievability for n→∞ and ϵ→0.

### Appendix A.3. Proof of Lemma 1

For any fixed $C_{B}\leq \left(N-1\right)C_{\mathrm{full}}\left(N\right)$, we first consider a realization of $\underline{\beta }$. Since $I_{m}\leq I_{m+1}$ for any m∈[1,N−1], by distributing $C_{B}$ on top of ${\left\{I_{m}\right\}}_{m=1}^{N-1}$ in a water-filling fashion, we can always have two possible cases if the equality of all terms on the RHS of (30) can not be achieved, i.e.,

(A18) $$\mathrm{Case}\;\left(a\right):\;\;\;L\left(C_{B},\underline{\beta }\right)<I_{j},$$

(A19) $$\;\mathrm{or}\;\;\;\;\;\;\mathrm{Case}\;\left(b\right):\;\;\;L\left(C_{B},\underline{\beta }\right)>I_{N},$$

where *j* is the smallest index such that $C_{m,m+1}=0$ for all m∈[j−1,N−1], and $L\left(C_{B},\underline{\beta }\right)=I_{m}+C_{m,m+1}$ for any *m* such that $C_{m,m+1}\neq 0$, namely the ‘water level’. Once the water-filling is performed, we fix the corresponding distribution of $C_{B}$.

For Case (a) where C=L, we now can decrease ${\left\{\beta_{m}\right\}}_{m=j}^{N}$ to increase $\beta_{1}$ such that L is increased. For Case (b) where $C=I_{N}$, we can look for an m<N with $\beta_{m}>0$ such that by decreasing $\beta_{m}$, $\beta_{N}$ increases and $I_{N}$ increases as well. Therefore, the $\underline{\beta }$ satisfying Case (a) and Case (b) are not optimal. The equality of the terms on the RHS of (30) is thus necessary.

### Appendix A.4. Proof of Proposition 2

Three steps are taken in the proof. In step (1) we show that an active compound mode 〈j,k〉 achieves the capacity if LB≤UB is satisfied. In step (2) we show that using any two separated active modes (all other modes are inactive) does not achieve the capacity. In step (3) we show that exact solutions of (54) and (55) are resulted.

**Step (1)** Note that function *g* given in (42) is convex-∩ when $\lambda \leq \frac{1}{k-1}$ if the largest activated mode is *k*. So, we set the partial derivatives of *g* with respect to ${\left\{\beta_{i}\right\}}_{i=1}^{N}$ according to the Kuhn–Tucker conditions, see ([23], eqn.4.4.10 and eqn.4.4.11), when the active *compound mode* 〈j,k〉 achieves the capacity. By considering (42) in nats, the partial derivative of function *g* with respect to $\beta_{i}$ is

(A20) $$\begin{array}{ccc} \frac{\partial g}{\partial \beta_{i}} & = & \frac{1-(k-1)\lambda }{2}\cdot \frac{iP}{1+\sum_{l=j}^{N}l\beta_{l}P}\\ \; & \; & +\frac{\lambda }{2}\sum_{m=i}^{N-1}\frac{iP}{1+\sum_{l=1}^{m}l\beta_{l}P}, \end{array}$$

where i∈[1:N].

**Step (1.1)** Firstly, by only considering that *compound mode* 〈j,k〉 is active, i.e., all ${\left\{\beta_{i}\right\}}_{i=j}^{k}$ are nonzero, while the other ${\left\{\beta_{i}\right\}}_{i=1}^{j-1}$ and ${\left\{\beta_{i}\right\}}_{i=k+1}^{N}$ are zeros, the partial derivative can be expressed as

(A21) $$\begin{array}{ccc} \frac{\partial g}{\partial \beta_{i}} & = & \frac{1-(k-1)\lambda }{2}\cdot \frac{iP}{1+\sum_{l=j}^{k}l\beta_{l}P}\\ \; & \; & +\frac{\lambda }{2}\sum_{m=i}^{k-1}\frac{iP}{1+\sum_{l=j}^{m}l\beta_{l}P}. \end{array}$$

For simplicity, we denote that

(A22) $$D\left(i\right)≜1+\sum_{l=j}^{i}l\beta_{l}P.$$

Now, consider that the partial derivatives corresponding to i∈[j:k] should be all identical to some value μ, i.e.,

(A23) $$\frac{\partial g}{\partial \beta_{j}}=\frac{\partial g}{\partial \beta_{j+1}}=\cdots =\frac{\partial g}{\partial \beta_{k}}:=\mu ,$$

to find the capacity solution in terms of optimal distribution of $\underline{\beta }$. Note that

(A24) $$\frac{\partial g}{\partial \beta_{k}}=\frac{1-(k-1)\lambda }{2}\cdot \frac{kP}{D(k)}=\mu .$$

For the case of k>j, we can recursively evaluate the equalities in (A23) as $\partial g/\partial \beta_{i}=\partial g/\partial \beta_{i-1}$ by taking *i* from *k* to j+1 in a descending order with the use of (A21). In such a way, it is obtained that

(A25) $$\frac{\lambda }{2}\cdot \frac{i(i+1)P}{D(i)}=\mu \;\;\;\mathrm{for}\;\;\;i\in \left[j:k-1\right].$$

Now, based on (A24) and (A25), we can derive expressions of ${\left\{\beta_{i}\right\}}_{i=j}^{k}$ by considering two scenarios.

Scenario 1: Consider k≥j+2, i.e., at least three consecutive modes are active. By taking i=j and i=j+1, (A25) can be used twice to obtain the equality $\frac{j}{D(j)}=\frac{j+2}{D(j+1)}$ that gives the relation

(A26) $$\beta_{j}P=\frac{\left(1+j\right)\beta_{j+1}P}{2}-\frac{1}{j}.$$

For k>j+2, expression (A25) allows us to further obtain

(A27) $$\frac{i(i+1)}{D(i)}=\frac{\left(i+1)(i+2\right)}{D(i+1)}$$

by taking *i* in the order of j+1 to k−1, which results in an interesting and important relation

(A28) $$\beta_{i}=\beta_{j+1}\;\;\;\;\mathrm{for}\;\;\;i\in \left[j+2:k-1\right].$$

Therefore, by applying relation (A26), we can express D(k) as

(A29) $$D\left(k\right)=\frac{k^{2}-k}{2}\beta_{j+1}P+k\beta_{k}P,$$

which is valid for the case of k=j+2 as well. So, by setting (A25) equal to (A24) with i=j as

(A30) $$\frac{1-(k-1)\lambda }{2}\cdot \frac{k}{D(k)}=\frac{\lambda }{2}\cdot \frac{j(j+1)}{D(j)},$$

and substituting D(k) in (A29), we can first derive the power of modes from j+1 to k−1 as

(A31) $$\beta_{j+1}P=\frac{2\lambda (1+jP)}{j(1-(j-1)\lambda )}.$$

According to (A26), we can then obtain the power of the first mode as

(A32) $$\beta_{j}P=\frac{\lambda (1+j)(1+jP)}{j(1-(j-1)\lambda )}-\frac{1}{j}.$$

Furthermore, owing to $\sum_{i=j}^{k}\beta_{i}=1$, we can finally represent the power of the last mode as

(A33) $$\beta_{k}P=P+\frac{1}{j}-\frac{1}{2}\left(2k-j-1\right)\beta_{j+1}P.$$

Now, applying the total power constraint, we should have

(A34) $$\left\{\begin{array}{l} 0<\beta_{k}P<P\\ 0<\beta_{j+1}P<P\\ 0<\beta_{j}P<P. \end{array}\right.$$

By substituting (A33), (A31), and (A32), the corresponding slope λ should simultaneously satisfy

(A35) $$\left\{\begin{array}{ccccc} \frac{1}{2(k-1)+jP(2k-j-1)} & < & \lambda & < & \frac{1}{2(k-1)}\\ 0 & < & \lambda & < & \frac{jP}{2+jP+j^{2}P}\\ \frac{1}{j(2+(j+1)P)} & < & \lambda & < & \frac{1}{2j}. \end{array}\right.$$

Since k>j+1, it is easy to see that the lower bound of λ is $\frac{1}{j(2+(j+1)P)}$. For the upper bound, if $\frac{1}{2(k-1)}>\frac{jP}{2+jP+j^{2}P}$, it leads to $P<\frac{2}{2jk-j^{2}-3j}$. Due to j≤k−2, such *P* results in

(A36) $$\begin{array}{c} j\left(2+\left(j+1\right)P\right)<2j+\frac{2(j+1)}{2k-j-3}\leq 2\left(k-1\right), \end{array}$$

which contradicts the lower bound. Therefore, to make the *compound mode* exist, the slope should be in the range

(A37) $$\frac{1}{j(2+(j+1)P)}<\lambda <\frac{1}{2(k-1)}.$$

Scenario 2: Consider k=j+1, i.e., the *compound mode* only consists of two *modes*. So, letting (A24) equal to (A25) gives the relation

(A38) $$\beta_{j}P=\frac{\lambda \left(1+j\right)\beta_{j+1}P}{1-2j\lambda }-\frac{1}{j}.$$

By considering $\beta_{j}+\beta_{j+1}=1$ now, it is obtained

(A39) $$\begin{array}{ccc} \beta_{j+1}P & = & \frac{\left(1-2\lambda j)(1+jP\right)}{j(1-(j-1)\lambda )} \end{array}$$

and a same $\beta_{j}P$ expression as in (A32). If such *compound mode* gives optimal solution, the condition $0<\beta_{j}P<P$ should be also satisfied, which gives $\lambda \in (\frac{1}{j(2+(1+j)P)},\frac{1}{2j})$ that is consistent with the result of (A37).

**Step (1.2)** Secondly, consider that the derivative $\partial g/\partial \beta_{k+i}$ for ∀i∈[1:N−k] with k<N should be less than μ as denoted in Step (1.1). Since ${\left\{\beta_{k+i}\right\}}_{i=1}^{N-k}=0$, we have

(A40) $$\begin{array}{c} \frac{\partial g}{\partial \beta_{k+i}}=\frac{1-(k+i-1)\lambda }{2}\cdot \frac{(k+i)P}{D(k)} \end{array}$$

(A41) $$\begin{array}{cc} \leq & \frac{\partial g}{\partial \beta_{k}}=\frac{1-(k-1)\lambda }{2}\cdot \frac{kP}{D(k)} \end{array}$$

which directly gives

(A42) $$\lambda \geq \frac{1}{2k}.$$

**Step (1.3)** Finally, consider that the derivative $\partial g/\partial \beta_{j-i}$ for ∀i∈[1:j−1] with j>1 should be less than μ as well. Since ${\left\{\beta_{j-i}\right\}}_{i=1}^{j-1}=0$, we have

(A43) $$\frac{\partial g}{\partial \beta_{j-i}}=\frac{1-(k-1)\lambda }{2}\cdot \frac{(j-i)P}{D(k)}+\frac{\lambda }{2}\left(j-i\right)P+\frac{\lambda }{2}\sum_{m=j}^{k-1}\frac{(j-i)P}{D(m)}.$$

Similarly, by upper bounding this derivative by μ given in (A24), we can have

(A44) $$\frac{1-(k-1)\lambda }{2}\cdot \frac{iP}{D(k)}+\frac{\lambda }{2}\sum_{m=j}^{k-1}\frac{iP}{D(m)}\geq \frac{\lambda }{2}\left(j-i\right)P,$$

where the summation can be computed by using relation of (A25) as

(A45) $$\frac{\lambda }{2}\sum_{m=j}^{k-1}\frac{iP}{D(m)}=i\mu \left(\frac{1}{j}-\frac{1}{k}\right).$$

By further incorporating μ given by (A24) and D(k) given by (A29) into (A44), the inequality becomes to

(A46) $$\frac{1-(k-1)\lambda }{j}\geq \frac{(j-1)\lambda }{i}\left(\frac{k-1}{2}\beta_{j+1}P+\beta_{k}P\right).$$

By substituting (A31) and (A33) for $\beta_{j+1}P$ and $\beta_{k}P$, it can be easily shown that

(A47) $$\lambda \leq \frac{i}{i(j-i)+(j-1)(1+jP)}\leq \frac{1}{\left(j-1)(2+jP\right)},$$

where i≥1 is applied in the bounding for the last step.

Note that, Step (1.2) and (1.3) and resulted bounds of λ also cover the the case of j=k, i.e., only one mode is active and achieves the capacity. Now, by considering the ranges given by (A37), (A42), and (A47), the slope bounds in (52) and (53) are resulted.

**Step (2)** Assume that the capacity can also be achieved by only activating any two separated modes $j^{\prime }$ and $k^{\prime }$, where $j^{\prime }\in \left[1:k^{\prime }-1\right]$ and $k^{\prime }\in \left[j^{\prime }+1,N\right]$. Then, the Kuhn–Tucker condition requires $\partial g/\beta_{k^{\prime }}=\partial g/\beta_{j^{\prime }}$, which results in the relation of

(A48) $$\frac{1-(k^{\prime }-1)\lambda }{2}\frac{1}{j^{\prime }D\left(k^{\prime }\right)}=\frac{\lambda }{2}\frac{1}{1+j^{\prime }\beta_{j}^{\prime }}.$$

Note that in $D(k^{\prime })$ only $\beta_{k}^{\prime }$ and $\beta_{j}^{\prime }$ are nonzero. Moreover, for $\forall i\in [1:k^{\prime }-j^{\prime }-1]$, we have

(A49) $$\frac{\partial g}{\partial \beta_{k^{\prime }-i}}=\frac{1-(k^{\prime }-1)\lambda }{2}\frac{(k^{\prime }-i)P}{D(k^{\prime })}+\frac{\lambda }{2}\frac{i(k^{\prime }-i)P}{1+j^{\prime }\beta_{j}^{\prime }}$$

By substituting the relation (A48) into above derivative, it can be shown that

(A50) $$\begin{array}{ccc} \partial g/\beta_{k^{\prime }-i}-\partial g/\beta_{k}^{\prime } & = & P\frac{1-(k^{\prime }-1)\lambda }{2D(k^{\prime })}\left(k^{\prime }-i+\frac{i(k^{\prime }-i)}{j^{\prime }}-k^{\prime }\right)\\ \; & = & P\frac{1-(k^{\prime }-1)\lambda }{2D(k^{\prime })}i\left(\frac{k^{\prime }-i}{j^{\prime }}-1\right)\\ \; & > & 0 \end{array}$$

where the last step is due to $k^{\prime }-i>j^{\prime }$. This result contradicts to the Kuhn–Tucker condition. This demonstrates the only compound modes achieves the capacity.

**Step (3)** For a slope λ in the range (52) and (53), the obtained optimal $\underline{\beta }$ from Step (1.1) can be used to evaluate (38) and (39) directly. Thus, (54) and (55) are resulted, respectively.

### Appendix A.5. Proof of Corollary 3

Consider four possible settings of UB and LB based on (52) and (53) as

$$\begin{array}{ccc} \mathrm{Case}\;1:\frac{1}{2(k-1)}\; & \leq & \;b\left(j-1\right),\;\;\frac{1}{2k}\leq b\left(j\right),\;\;b\left(j\right)<\frac{1}{2(k-1)},\\ \mathrm{Case}\;2:\frac{1}{2(k-1)}\; & \geq & \;b\left(j-1\right),\;\;\frac{1}{2k}\leq b\left(j\right),\;\;b\left(j\right)<b\left(j-1\right),\\ \mathrm{Case}\;3:\frac{1}{2(k-1)}\; & \leq & \;b\left(j-1\right),\;\;\frac{1}{2k}\geq b\left(j\right),\;\;\frac{1}{2k}<\frac{1}{2(k-1)},\\ \mathrm{Case}\;4:\frac{1}{2(k-1)}\; & \geq & \;b\left(j-1\right),\;\;\frac{1}{2k}\geq b\left(j\right),\;\;\frac{1}{2k}\leq b\left(j-1\right), \end{array}$$

where inequalities given by $\frac{1}{2k}$ are only valid for k<N. By evaluating the inequalities, the power condition (57) is resulted. Since the LB and UB are derived from the Kuhn–Tucker conditions, the derived power condition is a sufficient and necessary condition. This result directly gives that using compound mode $\langle j^{\prime },k^{\prime }\rangle$ with $j^{\prime }>j$ and $k^{\prime }<k$ is not a capacity solution.

### Appendix A.6. Proof of Lemma 2

By following the method in [20], we define f(x)=x−ln(x+1) and prove

(A51) $$f\left(x\right)+\sqrt[{3}]{2f(x)}-x\geq 0$$

for x≥0. Once this is done, one can take the procedure applied in ([20], Thm. 1) and the lower bound (61) follows.

Now, we start converting (A51) to an equivalent problem. First, by substituting f(x) in (A51), we need to prove

(A52) $$\sqrt[{3}]{2x-2\ln(1+x)}\geq \ln\left(1+x\right).$$

By denoting t:=x+1 with t≥1 and considering $x^{3}$ and $x^{\frac{1}{3}}$ are monotonically increasing in *x* for x≥0, showing (A52) is equivalent to show

(A53) $$2t-2-\ln t-{\left(\ln t\right)}^{3}\geq 0.$$

let $v\left(t\right):=2t-2-\ln t-{\left(\ln t\right)}^{3}$, we can focus on showing

(A54) $$v^{\prime }\left(t\right)=2-2t^{-1}-3t^{-1}{\left(\ln t\right)}^{2}\geq 0,$$

since v(1)=0. Considering t≥1, we finally convert proving (A51) to demonstrating

(A55) $$\psi \left(t\right):=tv^{\prime }\left(t\right)=2t-2-3{\left(\ln t\right)}^{2}\geq 0.$$

To do so, we take the first derivative of ψ(t) and set it to zero to have

(A56) 3lnt=t.

By solving (A56), we can evaluate the local maxima and/or local minima of ψ(t). It can be seen that (A56) has only two real roots for t≥1, which are

(A57) $$t_{1}=-3W_{0}\left(-\frac{1}{3}\right)\;\;\;\mathrm{and}\;\;\;t_{2}=-3W_{-1}\left(-\frac{1}{3}\right),$$

where $W_{0}\left(\cdot \right)$ is the principle branch of the Lambert *W* function defined over $\left[-e^{-1},\infty \right)$. Based on the property of the Lambert *W* function, $1<t_{1}<t_{2}$. Therefore, since ψ(1)=0, we can have two scenarios as

- Scenario 1: if $\psi (t_{1})>0$, we must have $\psi \left(t_{2}\right)<\psi \left(t_{1}\right)$, i.e., $t_{1}$ gives a local maxima and $t_{2}$ gives a minima;
- Scenario 2: if $\psi (t_{1})<0$, we must have $\psi \left(t_{2}\right)>\psi \left(t_{1}\right)$, i.e., $t_{1}$ gives a global minima and $t_{2}$ gives a maxima.

Hence, if we can prove that $\psi (t_{1})>0$ and $\psi (t_{2})>0$, both, we can conclude (A55) and thus (A51). We show this in what follows.

By setting $t^{⋆}=t_{1}\;or\;t_{2}$, $\psi (t^{⋆})$ can be expressed as

(A58) $$\psi \left(t^{⋆}\right)=\psi \left(t\right){|}_{t_{1},t_{2}}=2t^{⋆}-2-\frac{{\left(t^{⋆}\right)}^{2}}{3}.$$

If $\psi (t^{⋆})>0$, it is equivalent to have ${\left(t^{⋆}\right)}^{2}-6t^{⋆}+6<0$, which results in the range of

(A59) $$3-\sqrt{3}<t^{⋆}<3+\sqrt{3}$$

must be satisfied. For $t_{2}$, by applying the bounds given in ([20], Thm. 1) which is $-1-\sqrt{2x}-x<W_{-1}\left(-e^{-x-1}\right)<-1-\sqrt{2x}-\frac{2}{3}x$, it is easily obtained that $4.53<t_{2}<4.62$, which is in the range (A59). So, $\psi (t_{2})>0$ follows. For $t_{1}$, we apply an upper bound on $W_{0}\left(\cdot \right)$ given in ([24], Thm. 2.3), which is

(A60) $$W_{0}\left(x\right)\leq \ln\left(\frac{x+y}{1+\ln y}\right)$$

for $x>-e^{-1}$ and $y>e^{-1}$. By taking y=0.5, $t_{1}$ can be bounded as $t_{1}>1.8$. By also considering $t_{1}<t_{2}$, $t_{1}$ must in the range (A59) as well, which completes showing $\psi (t_{1})>0$.

Numerical evaluation of the bound (61) verifies the proof as illustrated in Figure A3, where the bound in [20] is plotted as a reference as well.
